# Croconaine-based NIR-II fluorescence imaging-guided tumor photothermal therapy induces long-term antitumor immune memory

**DOI:** 10.1186/s12951-024-02695-y

**Published:** 2024-08-13

**Authors:** Yafang Dong, Huifang Wang, Xiaodong Zhang, Youbin Ding, Yucheng Zou, Jigang Wang, Shan-Chao Zhao, Zhijie Li

**Affiliations:** 1 Department of Urology, Guangdong Provincial Clinical Research Center for Geriatrics, Shenzhen Clinical Research Center for Geriatric, Shenzhen People’s Hospital, The First Affiliated Hospital, Southern University of Science and Technology, Shenzhen, Guangdong 518020 P. R. China; 2 Department of Urology, the Third Affiliated Hospital of Southern Medical University, Guangzhou, Guangdong 510500 P. R. China; 3 Department of Medical Imaging, The Third Affiliated Hospital of Southern Medical University, Guangzhou, Guangdong 510630 P. R. China; 4 Department of Urology, the Fifth Affiliated Hospital, Southern Medical University, Guangzhou, Guangdong 510500 P. R. China; 5 Department of Urology, Nanfang Hospital, Southern Medical University, Guangzhou, Guangdong 510515 P. R. China; 6 Department of Traditional Chinese Medicine, School of Pharmaceutical Sciences, Southern Medical University, Guangzhou, Guangdong 510515 P. R. China; 7 State Key Laboratory for Quality Ensurance and Sustainable Use of Dao-di Herbs, Artemisinin Research Center, Institute of Chinese Materia Medica, China Academy of Chinese Medical Sciences, Beijing, 100700 P. R. China; 8 State Key Laboratory of Antiviral Drugs, School of Pharmacy, Henan University, Kaifeng, 475004 China; 9 Department of Oncology, the Affiliated Hospital of Southwest Medical University, Luzhou, Sichuan P. R. China

**Keywords:** Organic small molecule photothermal agents, Croconaine dyes, NIR II fluorescence imaging, Photothermal therapy (PTT), Imaging-guided photothermal therapy, Antitumor immune

## Abstract

Photothermal therapy (PTT) for cancers guided by optical imaging has recently shown great potential for precise diagnosis and efficient therapy. The second near-infrared window (NIR-II, 1000–1700 nm) fluorescence imaging (FLI) is highly desirable owing to its good spatial and temporal resolution, deep tissue penetration, and negligible tissue toxicity. Organic small molecules are attractive as imaging and treatment agents in biomedical research because of their low toxicity, fast clearance rate, diverse structures, ease of modification, and excellent biocompatibility. Various organic small molecules have been investigated for biomedical applications. However, there are few reports on the use of croconaine dyes (CRs), especially NIR-II emission CRs. To our knowledge, there have been no prior reports of NIR-II emissive small organic photothermal agents (SOPTAs) based on CRs. Herein, we report a croconaine dye (CR-TPE-T)-based nanoparticle (CR NP) with absorption and fluorescence emission in the NIR-I and NIR-II windows, respectively. The CR NPs exhibited intense NIR absorption, outstanding photothermal properties, and good biological compatibility. In vivo studies showed that CR NPs not only achieved real-time, noninvasive NIR-II FLI of tumors, but also induced significant tumor ablation with laser irradiation guided by imaging, without apparent side effects, and promoted the formation of antitumor immune memory in a colorectal cancer model. In addition, the CR NPs displayed efficient inhibition of breast tumor growth, improved longevity of mice and triggered efficient systemic immune responses, which further inhibited tumor metastasis to the lungs. Our study demonstrates the great potential of CRs as therapeutic agents in the NIR-II region for cancer diagnosis.

**Supplementary Information:**

The online version contains supplementary material available at 10.1186/s12951-024-02695-y.

## Introduction

Cancer is one of the main leading causes of death in the world [1–3]. Therefore, there is a need to develop improved techniques for early detection and treatment of cancer. Phototheranostics (photodiagnosis-guided phototherapy), which provides simultaneous disease diagnosis and therapy using light irradiation, is noninvasive and highly efficient, and has been shown to be an effective cancer treatment modality [4, 5]. Fluorescence imaging (FLI) can be used to visualize biological events with high sensitivity/spatial resolution, and noninvasiveness, and has drawn much interest in the field of biomedicine [6]. Of particular interest, second near-infrared (NIR-II, 1000–1700 nm) FLI revealed diminished photoscattering and negligible autofluorescence, enables substantial tissue penetration and great spatial resolution, and has been extensively explored to examine the tumor heterogeneity and progression over the past decade [7–9].

Photothermal therapy (PTT), a major category of phototherapy, is a promising and highly potent therapeutic approach for various diseases and cancers, characterized by noninvasiveness, high selectivity, and limited side effects [10]. PTT uses photothermal agents (PTAs) to convert NIR light energy into localized heat, leading to tumor immunogenic cell death (ICD), apoptosis, and necrosis [11, 12]. PTAs, which are essential elements of PTT, are usually nanomaterials or small molecules encapsulated in nanoparticles (NPs) [13–15]. Those NPs exhibit unique properties that enable precise delivery of various therapeutic agents and monitoring of tissue cellular responses, resulting in precise and efficient PTT [16]. The photothermal conversion efficiency (PCE) and biocompatibility of PTAs directly determine the antitumor effects of PTT. Enhanced PCE increases the suppression of tumor cell proliferation, thereby boosting the treatment survival rate [17]. To date, many inorganic nanomaterial-based PTAs, such as noble metal NPs [18, 19], carbon-based nanostructures [20, 21], and organic material-based PTAs [22, 23], have been developed to improve PTT efficiency. Inorganic agents have the drawbacks of potential long-term safety concerns, poor biodegradation, and unclear metabolic pathways, which restrict their clinical applications [6, 22]. Organic polymeric materials typically absorb radiation in the NIR-II window; however, their poor solubility and metabolism restrict their clinical applications [24]. In contrast, organic small-molecule PTAs (SOPTAs) exhibit extraordinary advantages in disease treatment because of their finely adjusted molecular structures and optical properties, outstanding biocompatibility and biodegradability, and excellent reproducibility [22, 25, 26]. Therefore, SOPTAs have been proposed as the most promising type of PTAs.

In recent decades, major advancements have been made in the synthesis and development of effective SOPTAs for PTT [22, 23]. To date, a number of SOPTAs including benzobisthiadiazole (BBTD), triazole [4,5-g]-quinoxaline (TQ), polymethine dyes (cyanines, squaraines, and croconaine dyes), dipyrromethene boron difluoride (BODIPY)/aza-BODIPY, and diketopyrrolopyrrole (DPP)-based molecules have been developed [22]. Currently, imaging-guided therapy, especially NIR-II FLI-guided PTT, is recognized as a viable approach to concurrently diagnose and treat diseases, particularly in cancer theranostics [27–29]. However, studies on NIR-II-based imaging-guided cancer phototherapy are still in their infancy.

Croconaine dyes (CRs) are zwitterionic compounds with a five-membered plane-rigid ring in the middle of the backbone structure [30]. The extended conjugation of CRs strongly promotes the resonance of electrons and the transfer of energy, providing intense and sharp NIR absorption, high PCE, and excellent chemical, thermal, and photostability [31, 32]. In particular, CRs can be readily synthesized and structurally modified, making them potential candidates for phototheranostic applications [30, 33]. To date, there have been several reports on CRs as photoacoustic imaging (PAI) or PTT agents [34–39]; however, few reports are available on CRs with NIR-II emission. Hence, appropriate molecular designs are critically needed to endow CRs with long-wavelength emissions.

**Scheme 1:**
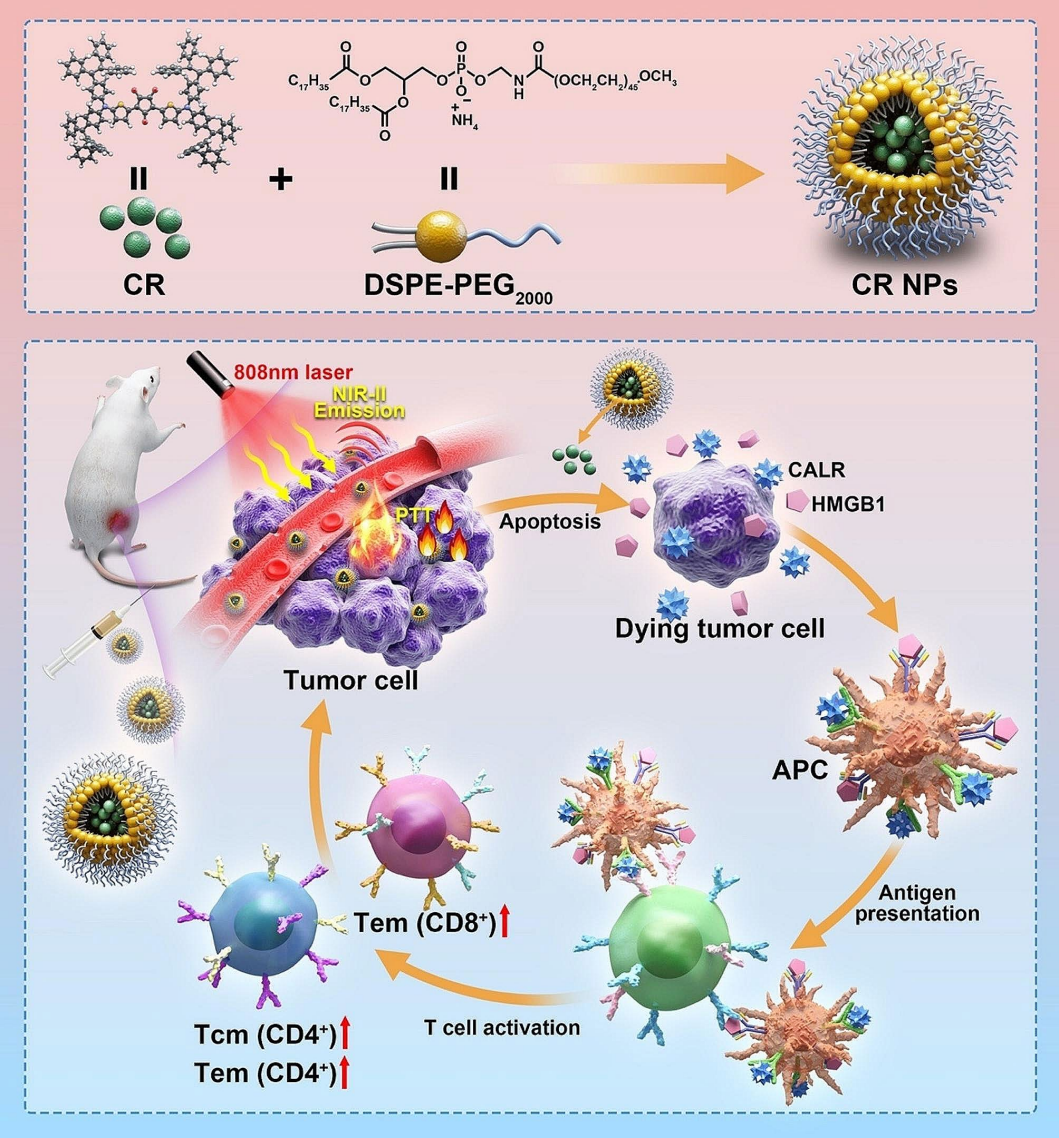
Graphical depiction of croconaine dye nanoparticles (CR NPs) for tumor-targeting photothermal therapy (PTT) guided by near-infrared II (NIR-II) fluorescent imaging (FLI). APC, antigen-presenting cells; CR, croconaine dye; DSPE, distearoylphosphatidylethanolamine; NPs, nanoparticles; PEG, polyethylene glycol

Inspired by the above findings, we synthesized a croconaine dye-based compound [32], CR-TPE-T, which showed NIR-I absorption and NIR-II emission, for theranostic (NIR-II FLI and PTT) applications. Two electron donors (D) and a strong electron acceptor (A) were joined to construct the “D − A−D” structure, CR-TPE-T. CR-encapuslated nanoparticles (CR NPs) were formed by assembling hydrophobic CR-TPE-T into an amphiphilic biocompatible copolymer (DSPE-mPEG_2000_) using a simple nanoprecipitation approach. The resulting NPs displayed strong NIR absorption (600–1050 nm), prominent photothermal conversion capacity, acceptable fluorescence, good size and photo-stabilities and excellent biocompatibility in aqueous solution. An in vivo study revealed that the CR NPs could effectively accumulate at the tumor site through the enhanced permeability and retention (EPR) effect, which can be visualized by NIR-II FLI. Moreover, benefiting from the NIR-II FLI-guided PTT, CR NPs can also achieve strong antitumor effects using 808-nm laser irradiation and promote the formation of immune memory in a colorectal cancer model (Scheme 1). Notably, the excellent in vivo antitumor performance was obtained simply through a single injection and laser treatment. The excellent in vivo biosafety of CR NPs was also evaluated by histopathological and hematologic analysis. Moreover, CR NPs displayed efficient photothermal effects in 4T1 breast tumor-bearing mice, and inhibited tumor growth, improved longevity, and induced effective systemic immune responses, leading to heightened suppression of lung metastasis. To our knowledge, this is the first study of a CR-based NIR-II FLI/PTT organic small molecule. This study enriches the diversity of SOPTAs with NIR-II emission, facilitating the development of NIR-II FLI-guided PTT for multifunctional biomedical applications.

## Methodology

### Preparation of CR NPs and fluorescent dye (DIR) loaded nanoparticles

CR NPs were fabricated through a widely used nanoprecipitation approach. 0.5 mL THF solution of CR (1.0 mg mL^− 1^) and 0.5 mL DSPE-PEG2000 (20 mg mL^− 1^) were mixed homogeneously via ultrasound for 5 min. Afterward, the above solution was dropwise injected into 2 mL of Milli-Q water under sonication. The resulting mixture was further continuative sonication for 20 min. The residue THF solvent was removed with a rotary evaporator. The as-prepared CR NPs were then filtered by a 0.22 μm syringe-driven filter, and concentrated by centrifugation- ultrafiltration for future use.

The fluorescent dye-loaded CR NPs, CR/DIR NPs, were manufactured using the same method as the CR NPs. A mixture of THF solution: DIR (1.0 mg mL^− 1^, 0.1 mL) CR (1.0 mg mL^− 1^, 0.4 mL) and DSPE-PEG2000 (20 mg mL^− 1^, 0.5 mL) were used.

### Quantitative measurement of CR encapsulated into CR NPs

The calculation was based on a previous report [26]. The amount of CR encapsulated in the CR NPs was measured by UV-Vis absorption spectroscopy. The calibration curve was linear with a concentration in the range of 5–100 µg mL^− 1^. As a result, the concentration of CR in the CR NPs was 44.3 µg ml^− 1^, thus the encapsulation efficiency of CR in NPs was 89%. [44.3 *5 / 250 *100% = 89%]

### Size, zeta potential and morphology of CR NPs

Dynamic light scattering (DLS) was used to measure the size and zeta potential, which was performed on Zetasizer Nano ZS90 (Malvern). 1.0 mL of 50 µg mL^− 1^ CR NPs aqueous solution was used. The morphology of CR NPs was observed using Transmission electron microscopy (TEM, JEOL, JEM-F200).

### Fluorescence quantum yield measurements of CR NPs

The fluorescence quantum yield (QY) of CR NPs was measured utilizing dye IR-1061 as a reference, whose QY has been reported as (1.7 ± 0.5) % in DCM [40]. The QY value of CR NPs was measured based on four concentrations with optical density (OD) of 0.018, 0.050, 0.074 and 0.099 at 808 nm. The integrated total emission intensity was in the 900–1300 nm region. Two slopes, one obtained for the IR-1061 reference and the other from CR NPs, were used in the calculation of the quantum yield of CR NPs in water, according to the following equation [41].

$$Q{Y_{{\rm{sample}}}} = Q{Y_{{\rm{ref}}}} \times \frac{{slop{e_{_{{\rm{sample}}}}}}}{{slop{e_{_{{\rm{ref}}}}}}} \times \frac{{\eta _{{\rm{sample}}}^2}}{{\eta _{ref}^2}}$$

where *η*_*sample*_ and *η*_*ref*_ are the refractive index of water and DCM, respectively.

### Singlet oxygen detection

CR NPs aqueous solution (200 µg mL^− 1^, 100 µL) and DPBF (50 µM in DMF, 100 µL) were added into water in a quartz cuvette and then irradiated (808 nm, 1 W cm^− 2^) for 180s, the absorption spectra of the DPBF were examined every 30s.

### Photothermal properties of CR NPs

For the purpose of investigating the photothermal properties of CR NPs, CR NPs (50 µg mL^− 1^) was irradiated with 808 nm laser at different power densities (0.2–1.2 W cm^− 2^). The temperature changes across differing doses (25, 50, 100, 150 and 200 µg mL^− 1^) of CR NPs upon exposure to the laser (0.8 W cm^− 2^, 6 min) were also recorded. The temperature changes were recorded with an IR thermal camera. For the photothermal stability, sample temperature was recorded during five heating/cooling progresses. In one cycle, NIR laser was initially irradiate the sample five minutes, then left to cool down for five minutes.

### Cellular culturing

Colon 26 and 4T1 cells were grown in RPMI1640 (Gibco) containing 10% (v/v) FBS (Gibco, Australia) plus 1% (v/v) antibiotics (HyClone). All cells were grown in an incubator at an environment of 5% CO_2_ (37 °C). A hemocytometer was utilized to check the density of cells and this was performed prior to any experiments.

### In vitro cytotoxicity

The cytotoxicity and photothermal ablation efficiency of CR NPs on Colon 26 cells or 4T1 cells were evaluated through a standard cell counting kit-8 (CCK-8) assay. Briefly, Colon 26 cells or 4T1 cells were seeded in 96-well plates (1 × 10^4^ cells/well) with six duplicate wells of each group and incubated in a standard cell culture atmosphere for 24 h. Then, different concentrations (0, 2.5, 5, 10, 20 and 40 µg mL^− 1^) of CR NPs in new culture medium was added, followed by another 12 h incubation. Subsequently, the cells were handled with irradiation (808 nm, 1.0 W cm^− 2^) for 5 min. Meanwhile, the cells without laser irradiation were also carried out for the study of dark cytotoxicity. Post-12-hour-incubating, the cells were then incubated with 10% CCK-8 (10 µL) for 2 h. Thereafter, cell viability of Colon 26 was measured through a microplate (450 nm, LabServ™K3, Thermo Fisher Scientific). The cell viability was calculated as follow formula: Cell viability (%) = (OD_sample_ - OD_background_)/(OD_control_ - OD_background_) × 100% [8, 42].

### Live/death cell-staining

Live/dead cell staining of Colon 26 cells was identified with the calcein acetoxymethyl ester (calcein AM) and propidium iodide (PI). The cells were seeded in 6-well plates (1 × 10^5^ cells/well), with 24 h of acclimatization under dark conditions before treatment. Cells were consequently exposed as described here: 100 µL PBS; 100 µL PBS plus laser irradiation (808 nm, 1.0 Wcm^− 2^, 5 min); 10 µg mL^− 1^ CR NPs; 10 µg mL^− 1^ CR NPs plus laser irradiation (808 nm, 1.0 Wcm^− 2^, 5 min). After a further 4 h incubation, the cells were rinsed thrice with PBS, and successively stained with 4 µL calcein-AM (1.0 mg mL^− 1^, live cells, green fluorescence) and 6 µL PI (1.0 mg mL^− 1^, dead cells, red fluorescence) for 30 min. Finally, the cells were gently washed and then exposed to imaging through confocal laser scanning microscopy.

### Flow cytometry experiments

CR NPs-mediated cell apoptosis was investigated using Annexin V-FITC/PI Apoptosis Detection Kit. In brief, Colon 26 cells were seeded and cultured into 6-well plates (1 × 10^5^ cells per well) for 24 h. Colon 26 cells were treated same with the live and dead cell staining and cultured for another 12 h. After treatments, Annexin V-FITC/PI was added to the cell culture for staining. Flow cytometry was used to test the cells.

### Animals and tumor model

All the in vivo experiments on the animals in this study were conducted according to the accepted regulations of the Institutional Animal Care and Use Committee (IACUC) of Shenzhen People’s hospital (AUP-240,115-LZJ-0001-01), and performed under legal protocols. The female BALB/c mice (≈ 20 g, 5–6-week, Guangzhou Gempharma Tech™) were used to build animal models. All animals were firstly acclimatized to the animal facility (Shenzhen Peoples hospital) for one week under Specific Pathogen Free (SPF) conditions prior to experimentation.

For Colon 26 tumor model, the PBS suspension of 1 × 10^6^ Colon 26 cells (100 µL) was subcutaneously transplanted in the right-back of each mouse. For lung metastasis model, 50 µL PBS suspension containing 5 × 10^5^ 4T1 cells was injected into the fourth mammary fat pad. The computational formula of the tumor volumes was (length × width^2^)/2. The subsequent in vivo investigations were conducted when tumor volume approached 100 mm^3^.

### Tumor fluorescence imaging of CR NPs in vivo and ex vivo

Colon 26 tumor-bearing mice were intravenously administered with CR/DIR NPs (100 µL, 50 µg mL^− 1^). The real-time fluorescent signals of mice at ddifferent timepoints (0, 1, 4, 8, 12, 24, 36 and 48 h) were recorded through IVIS Spectrum system. Fluorescence imaging was analyzed by LivingImage^®^ 4.3.1.

### In vivo NIR II fluorescence imaging and bio-distribution analysis of CR NPs

The performance of CR NPs for in vivo NIR-II fluorescence imaging was investigated on 4T1 tumor-bearing mice (*n* = 3). Briefly, the mice bearing 4T1 tumor were tail-intravenously injected with 100 µg of CR NPs (1.0 mg mL^− 1^) per mouse. Then, the real-time imaging was recorded and monitored at designated time intervals (0, 4, 8, 12, 24 and 36 h) post-injection through NIR-II in vivo imaging system MARS (Artemis Intelligent Imaging, Shanghai) (808 nm, 900 LP, power density 5000 mW cm^− 2^). For ex vivo biodistribution analysis, the mice for imaging were sacrificed at 36 h post-injection and their tumors and major organs (heart, liver, spleen, lung, kidney) were collected and imaged. The analysis of the signal intensity was performed by LightField software. The analysis of each image was conducted by Image J software.

### In vivo infrared thermography and phototherapeutic study

In order to investigate the in vivo anti-tumor ability of CR NPs, twenty Colon26 tumors bearing female mice with tumor volume approximately 100 mm^3^ were stochastically divided into four groups (*n* = 5). Such four groups were (1) only PBS, (2) PBS + laser (3) only CR NPs, (3) and (4) CR NPs + laser, and treatment as follows: tail vein injection of 200 µL PBS for (1) and (2) groups, CR NPs (1.0 mg mL^− 1^) for (3) and (4) groups. For laser irradiation groups (2) and (4), the tumors of mice were continuously exposed to irradiation of the 808 nm laser (1.0 W cm^− 2^) for 10 min at 12 h post-injection. During the irradiation process, infrared thermal images of whole bodies and real-time temperature variations of tumor areas were photographed and recorded using the infrared thermal imaging camera (the day received laser treatment was day 0).

### Histopathological and hematological analysis

For further biosafety evaluation, all mice were sacrificed at the end of therapy process and the tumors and major organs were collected. In addition, blood samples were harvested immediately for evaluation of blood biochemical parameters. The collected organs were fixed with 4% formalin solution and embedded in paraffin for hematoxylin-eosin (H&E) staining. Staining was analyzed through digital microscopy.

### Immunofluorescence staining

Cells were fixed with 2% paraformaldehyde, followed by incubation with a 3% BSA solution to minimize nonspecific binding. For intracellular staining, cells were permeabilized using a permeabilization buffer containing 0.3% Triton X-100. Next, the cells were exposed to primary antibodies, including anti-CALR and anti-HMGB1. After washing, the samples were incubated with fluorophore-conjugated secondary antibodies. Following washing steps, cells were stained with DAPI for nuclear visualization. Fluorescence imaging was conducted utilizing confocal laser scanning microscopy.

### Memory T cell analysis by flow cytometry

Spleens were aseptically harvested, minced, and homogenized through 70-µm cell strainers to obtain single-cell suspensions. These suspensions were then subjected to lysis using ACK buffer, followed by cell counting. Subsequently, 100 µL of the resulting suspension (1 × 10^6^) cells was incubated with anti-mouse CD16/CD32 antibody to block nonspecific binding. Afterward, cells were stained with fluorochrome-labeled antibodies including CD45-BV570, CD3-FITC, CD11b-APC-Cy7, CD4-APC, CD8a-PE, CD62L-BV421, and CD44-AF700 at 4 °C for 30 min. Following incubation, cells were washed and stained with ViaDye Red to identify dead cells. Finally, flow cytometric analysis was performed using a Cytek Aurora spectral flow cytometer, and data were analyzed using Flowjo software.

### Statistics

All the quantitative datasets are expressed as mean ± SEM (standard error of the mean) unless otherwise indicated. Statistical analyses of all the data were conducted by GraphPad Prism 8.3.1 software. *p* value ≤ 0.05 was deemed to confer statistical significance. Sample sizes were chosen based on guidance of literatures. Quantitative data were acquired from at least three replicate samples. ns: no significance. *p < 0.05, **p < 0.01, ***p < 0.001.

## Results and discussion

### Synthesis and characterization of the CR NPs

**Fig. 1:**
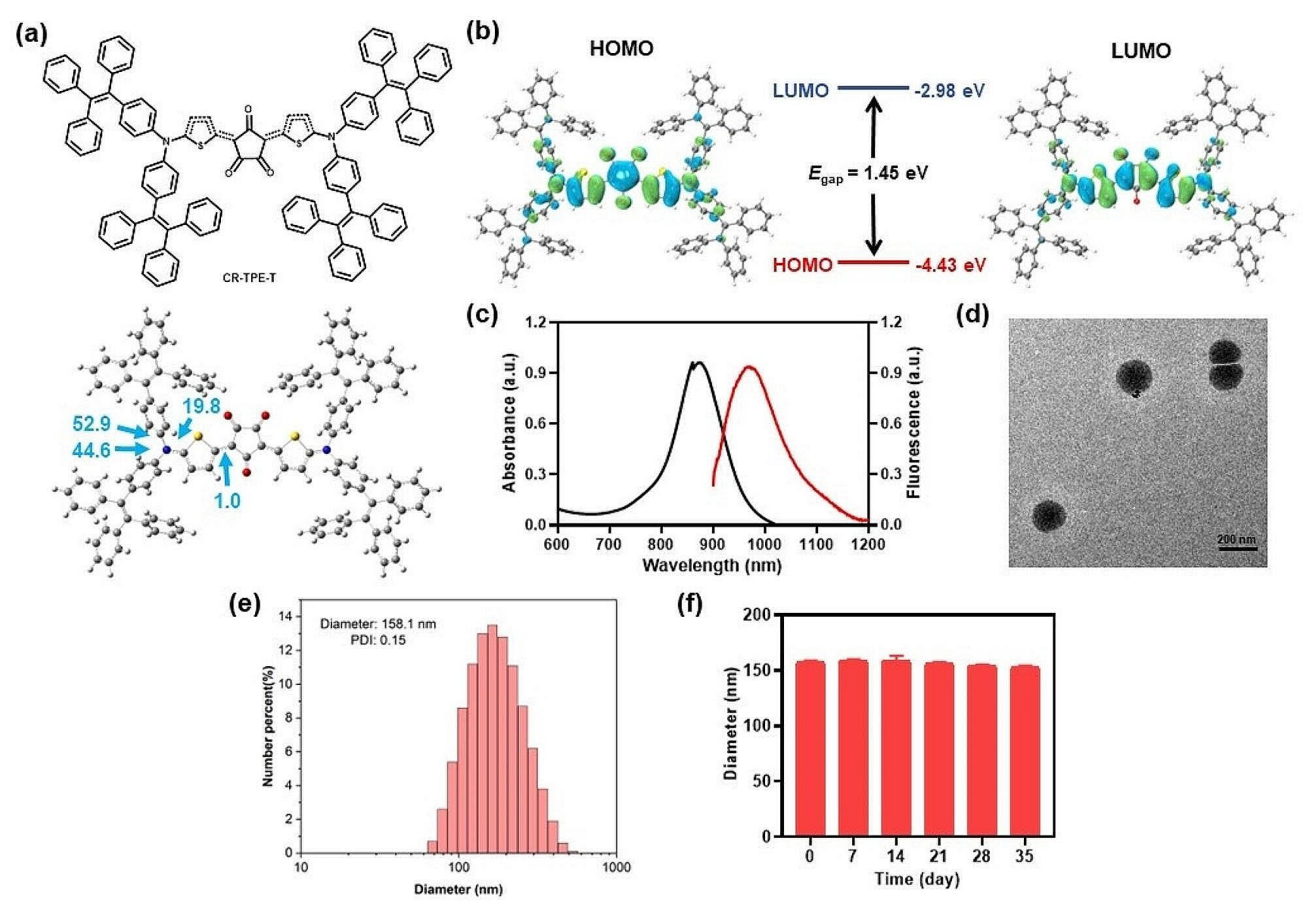
Characterization and properties of the CR NPs. (**a**) Chemical structure and optimized molecular geometry of CR-TPE-T. (**b**) The calculated frontier molecular orbitals of CR-TPE-T calculated by the density functional theory (DFT) calculation method at the B3LYP/6-31G level. (**c**) Absorption and fluorescent spectra (excited wavelength 808 nm) of croconaine dye nanoparticles (CR NPs) in aqueous solution. (**d**) Representative transmission electron microscopy (TEM) image and (**e**) Hydrodynamic size distribution of CR NPs. scale bar: 200 nm. Polydispersity index (PDI) = 0.15. (**f**) Size of CR NPs after 5 weeks of storage in the dark at room temperature

First, the D–π–A–π–D structured CR-TPE-T was synthesized through a single-step condensation between croconic acid and N, N-bis(4-(1,2,2-triphenylvinyl) phenyl) thiophen-2-amine in 1:2 equivalent based on the previous reports [32] (Scheme S1). The molecular geometry and electronic distribution of CR-TPE-T were studied by density functional theory (DFT) calculations. As shown in Fig. 1a, the optimized ground-state (S_0_) geometry of CR-TPE-T showed an axisymmetric configuration in space; the two dihedral angles between TPE and its neighboring thiophene ring were 52.9° and 44.6°. The dihedral angle between the thiophene ring and the croconic ring was 1.0°, and the whole molecule tended to appear as a planar conformation along the backbone. In CR-TPE-T, the electron densities of the highest occupied molecular orbital (HOMO) were well distributed on the tetraphenylethylene (TPE) donors along its conjugated thiophene units, whereas the lowest unoccupied molecular orbital (LUMO) was more delocalized on the electron-deficient croconaine core, revealing efficient intramolecular charge transfer within the molecule. The HOMO and LUMO energy bandgap of CR-TPE-T was approximately 1.45 eV (Fig. 1b).

For in vivo applications, the hydrophobic CR-TPE-T was then fabricated into nanoparticles (NPs) (CR NPs) through nanoprecipitation using DSPE-mPEG_2000_ as the encapsulation matrix. According to the normalized absorption spectrophotometer analysis, the calculated encapsulation efficiency of the CR NPs was 89% (Figure S1). The UV-vis-NIR absorption spectra analysis of the CR NPs displayed an intense characteristic absorption peak of 870 nm and extending over 1000 nm, matching very well with the biological window (700–1000 nm), which suggests that it has potential to serve as a PTA. In addition, the CR NPs showed the corresponding fluorescence emission covering the NIR-II region, which extends to 1200 nm, with maximum peak at 970 nm, the fluorescence quantum yield of CR NPs in water was calculated to be 0.47% by using IR 1061 as a reference (Figure S2), indicating the potential for NIR-II FLI (Fig. 1c).

The TEM image and DLS results in Fig. 1d and e indicated a dispersed spherical shape of CR NPs with average diameter of ∼ 158 nm, indicating that the NPs might achieve passive tumor accumulation through the EPR effect. Additionally, the CR NPs zeta potential was − 33.7 ± 1.71 mV, which favors good stability in a physiological environment (Figure S3). The colloidal stability of CR NPs was further studied by recording their particle size at room temperature for 5 weeks. Notably, negligible variation of the diameter distribution was observed for the NPs solution after storage at room temperature for 5 weeks, implying good colloidal stability of the CR NPs (Fig. 1f). These results demonstrate that CR NPs are a promising candidate for NIR-II FLI-guided PTT applications.

### In vitro NIR-II FLI and PTT properties of CR NPs

**Fig. 2:**
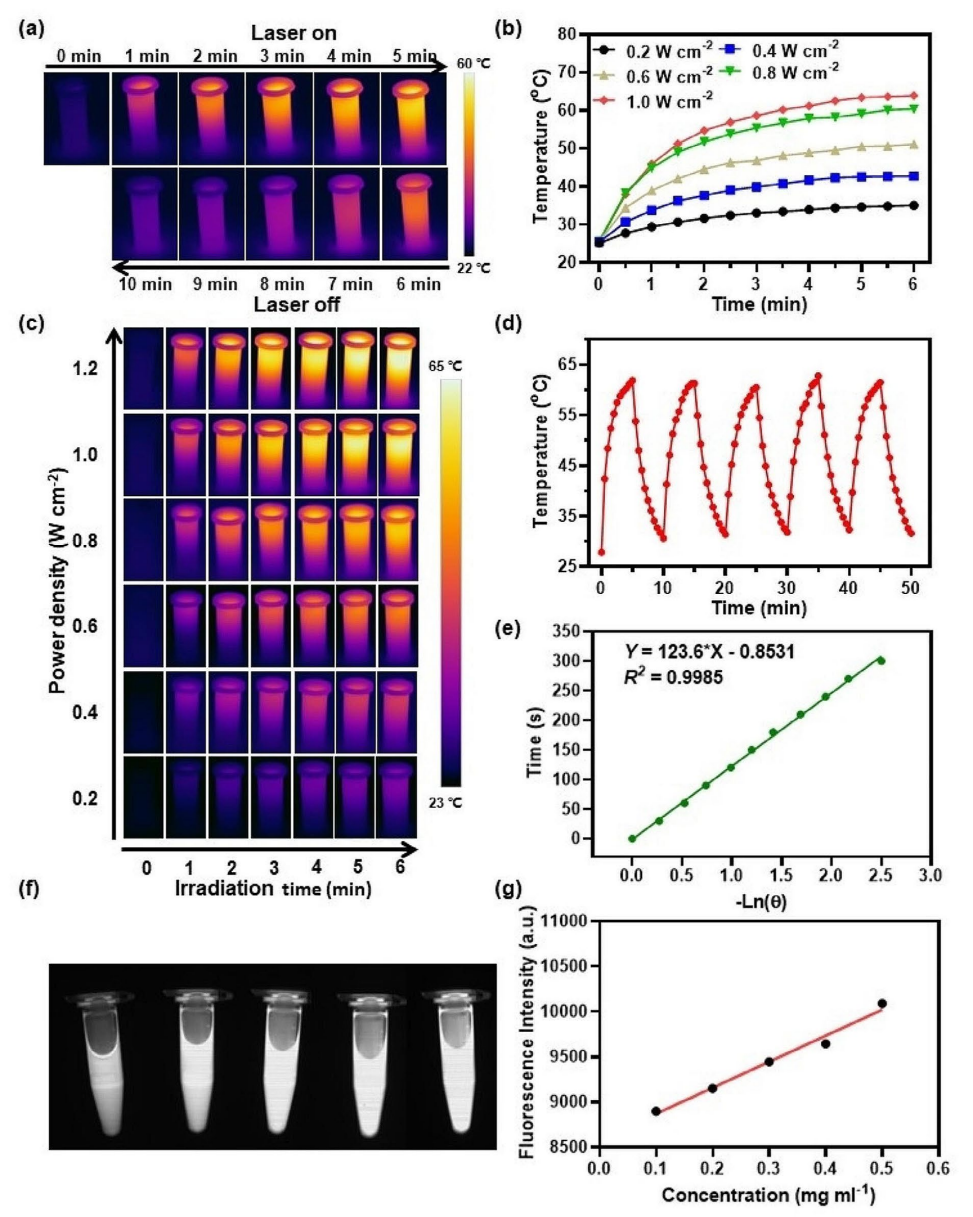
In vitro NIR-II FLI and PTT properties of CR NPs. (**a**) Infrared (IR) thermal images of 200 µg mL^–1^ CR NPs aqueous solution under 5 min irradiation (808 nm, 1.0 W cm^− 2^) followed by 5 min cooling period. (**b**) Photothermal performance and (**c**) real-time thermal imaging of CR NPs in aqueous solution with varied laser power densities (200 µg mL^–1^, 808 nm). (**d**) Photothermic stability of 200 µg mL^− 1^ CR NPs in aqueous solution (808 nm, 1.0 W cm^− 2^) during five successive laser ON/OFF cycles. (**e**) Time versus − Ln(θ) linear correlation derived from the cooling stage of Fig. 2d. (**f**) In vitro NIR-II signals of aqueous solution of CR NPs at different concentrations (0.1 − 0.5 mg mL^− 1^) under 808-nm excitation. (**g**) Quantitative relationship between fluorescence imaging (FLI) signal intensities and concentrations of CR NPs

Because of the inherent NIR absorption capacity of the CR NPs, we then investigated the PTT behavior of CR NPs in vitro. As shown in the infrared images (Fig. 2a) recorded by a thermal camera, the CR NPs exhibited an apparent photothermal effect on 808-nm laser irradiation. The laser power density effects on the temperature changes of CR NPs were then investigated. It was evident that the profiles of temperature increments of the CR NPs were positively related to laser power density (Fig. 2b). The temperature increased to approximately 62.5 °C at the power density of 1 W cm^− 2^ on 808-nm laser irradiation for 6 min, which was enough to kill tumors effectively. The CR NPs concentration (25 to 200 µg mL^− 1^) also showed a positive relationship with the photothermal effect at 1 W cm^− 2^ of laser power density and the maximum temperature was 64.3 °C (Figure S4b), whereas the control (phosphate-buffered saline [PBS]) group showed a negligible temperature change under identical conditions. The corresponding infrared (IR) thermographs confirmed the temperature changes (Fig. 2c and S4a). These results indicate controllable photothermal behavior. Photothermal stability is an important parameter for evaluating the capability of PTA. Then, the CR NPs were exposed to 808-nm laser over five continuous irradiation and cooling cycles to evaluate its photothermal stability (Fig. 2d). The highest temperature of the CR NPs in each cycle was remarkably consistent, even after 5 cycles of irradiation, implying the outstanding thermal- and photo-stability. Furthermore, on basis of the cooling curve (Fig. 2e) and the reported methods [43, 44], the estimated PCE of the CR NPs was 65%. To explore whether CR-NPs exhibit photodynamic effects, we studied the ROS production capability of CR NPs under 808 nm laser exposure. We used 1,3-diphenylisobenzofuran (DPBF) as an extracellular ^1^O_2_ trapper because it can be irreversibly oxidized by ^1^O_2_ [45]. As shown in Figure S5, the absorption intensity did not significantly decrease after laser irradiation, indicating no ^1^O_2_ was generated and, consequently, that CR-NPs do not exhibit photodynamic effects. Therefore, CR-NPs primarily function through photothermal effects without concurrent photodynamic activity.

Motivated by the outstanding optical performance of the CR NPs, we then studied the CR NPs’ in vitro NIR-II FLI performance using a 900-nm long-pass (LP) filter under 808-nm laser excitation. As depicted in Fig. 2f and g, the CR NPs showed strong NIR-II fluorescence in a concentration-dependent manner, with the fluorescence signal linearly correlated with its concentration in the range of 10 to 50 µg mL^− 1^, suggesting the feasibility of NIR-II bioimaging.

### In vitro antitumor performance evaluation of CR NPs

**Fig. 3:**
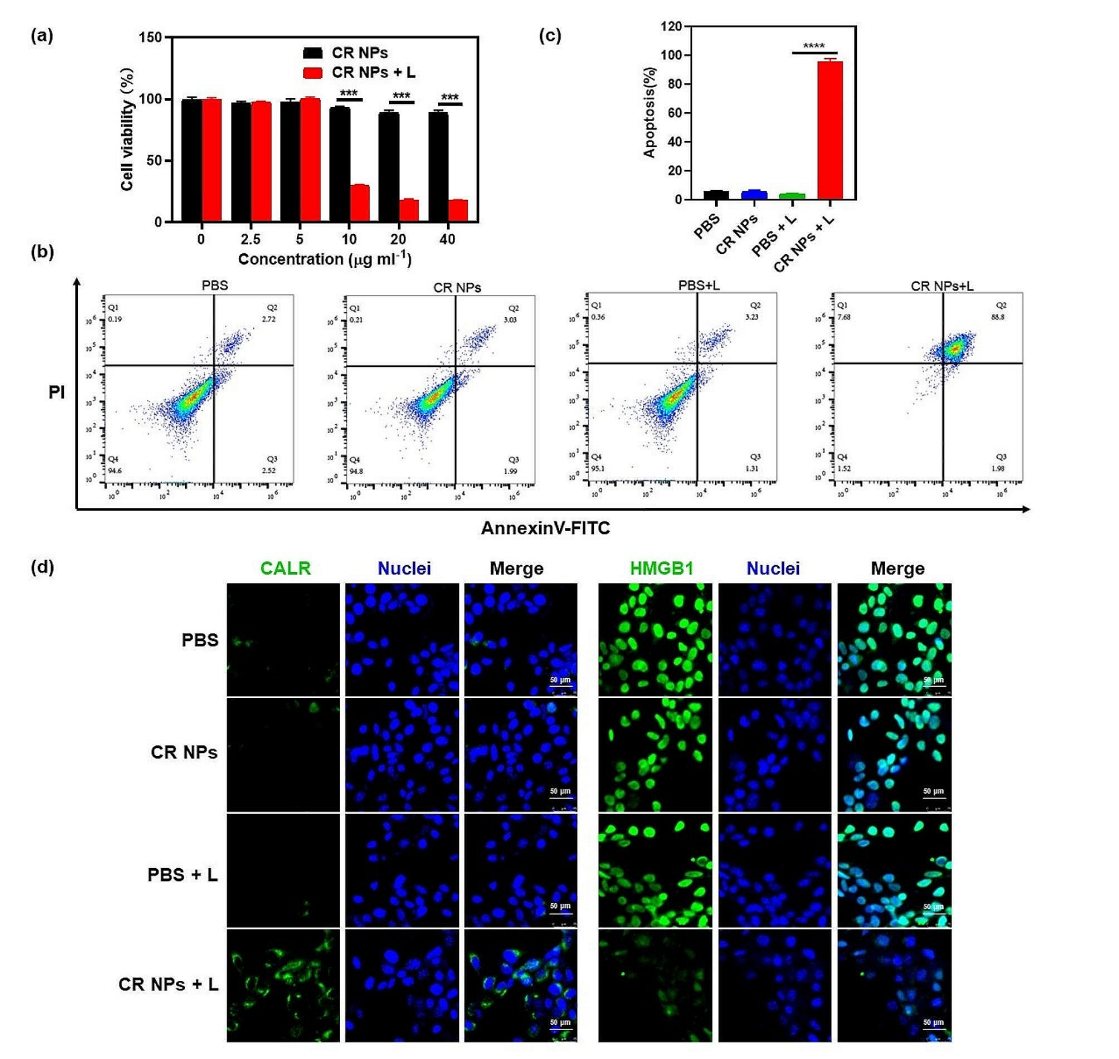
In vitro antitumor performance evaluation of CR NPs. (**a**) Relative Colon26 cell viability in relation to various CR NPs concentrations with or without laser irradiation, determined using a CCK-8 assay (*n* = 6). (**b**) Apoptosis analysis on Colon26 cells using flow cytometry after different treatments (CR NPs: 20 µg mL^− 1^). For (**a**) and (**b**), laser irradiation conditions: 808 nm, 1.0 W cm^− 2^, 5 min. (**c**) The corresponding quantitative analysis of (**b**). (**d**) Immunofluorescence staining of cell surface CALR and HMGB1 expression in various groups (*n* = 4). Scale bars = 50 μm

Given the superior photothermal properties of CR NPs, its PTT effectiveness in Colon26 and 4T1 was then studied (Fig. 3 and Figure S6). First, the photocytotoxicity of CR NPs was studied using a standard CCK-8 cell viability assay. As shown in Fig. 3a, without laser irradiation, the cell viability for Colon26 was negligibly influenced even the concentration of CR NPs was up to 40 µg mL^− 1^, revealing the excellent biocompatibility of CR NPs for biomedical and clinical applications. In contrast, significant photothermal cytotoxicity was observed after treatment with CR NPs + laser irradiation, and the cell viability decreased markedly with more than 80% of cells dying at a CR NP concentration of 20 µg mL^− 1^. Similar results were also obtained in 4T1 cells, showing that CR NPs + laser irradiation could significantly suppress the cell viability in a concentration-dependent manner (Figure S6a). Meanwhile, we noticed that 4T1 cells appeared to be more sensitive to CR NPs-induced photothermal effect than Colon26 since lower concentrations of CR NPs such as 2.5 and 5 µg ml^− 1^ have suggested prominent inhibition to viability upon irradiation. To further investigate the photothermal effect of CR NPs, we then conducted live (green fluorescence)/dead (red fluorescence) staining experiments. As shown in Figure S6b, cells treated with PBS, CR NPs and PBS + laser irradiation exhibited widespread green fluorescence, indicating that there was no anticancer effect in these groups. Red fluorescence was clearly observed in the CR NPs + laser irradiation group, and almost 70% of cells underwent death after irradiation. This illustrates the excellent PTT capacity of CR NPs against Colon26 cells, which is in line with CCK-8 findings. Furthermore, cell apoptosis was studied by flow cytometry. As expected, the CR NPs + laser group showed a remarkably high cell apoptotic ratio (∼95%), whereas, in the control groups, no obvious apoptotic or necrotic cells were observed, which further confirmed the high efficiency of the PTT effect of CR NPs (Fig. 3b and c). Together, CR NPs showed good biocompatibility and efficient PTT under laser irradiation, implying great potential for in vivo biomedical applications.

Studies have indicated that photothermal ablation of tumors could stimulate and redistribute immune cells, thereby triggering robust antitumor immune responses [46]. A crucial aspect of this immune activation is the induction of ICD. ICD occurs when tumor cells are subjected to external stimuli, mediating the body’s antitumor immune response [47]. ICD is accompanied by the production of a variety of damage-associated molecular patterns (DAMPs), such as the exposure of surface-exposed calreticulin (CALR) and the release of high-mobility group box 1 (HMGB1) [48–51]. Here, we investigated the expression of CALR on the cell surface and the release of HMGB1 in different groups. The PBS, CR NPs, and PBS + laser treatments showed little cell surface CALR exposure (green), whereas the CR NPs + laser treatment resulted in enhanced exposure of CALR on the cell surface owing to CR-mediated PTT under laser irradiation (Fig. 3d). In addition, the CR NPs + laser treatment group showed less HMGB1 staining in the cell nuclei compared with the other three groups, indicating a prominent release of HMGB1. Collectively, these results indicated that CR NPs can broadly induce tumor cell apoptosis and ICD after laser irradiation.

### In vivo NIR-II fluorescence imaging and biodistribution analysis of CR NPs

**Fig. 4:**
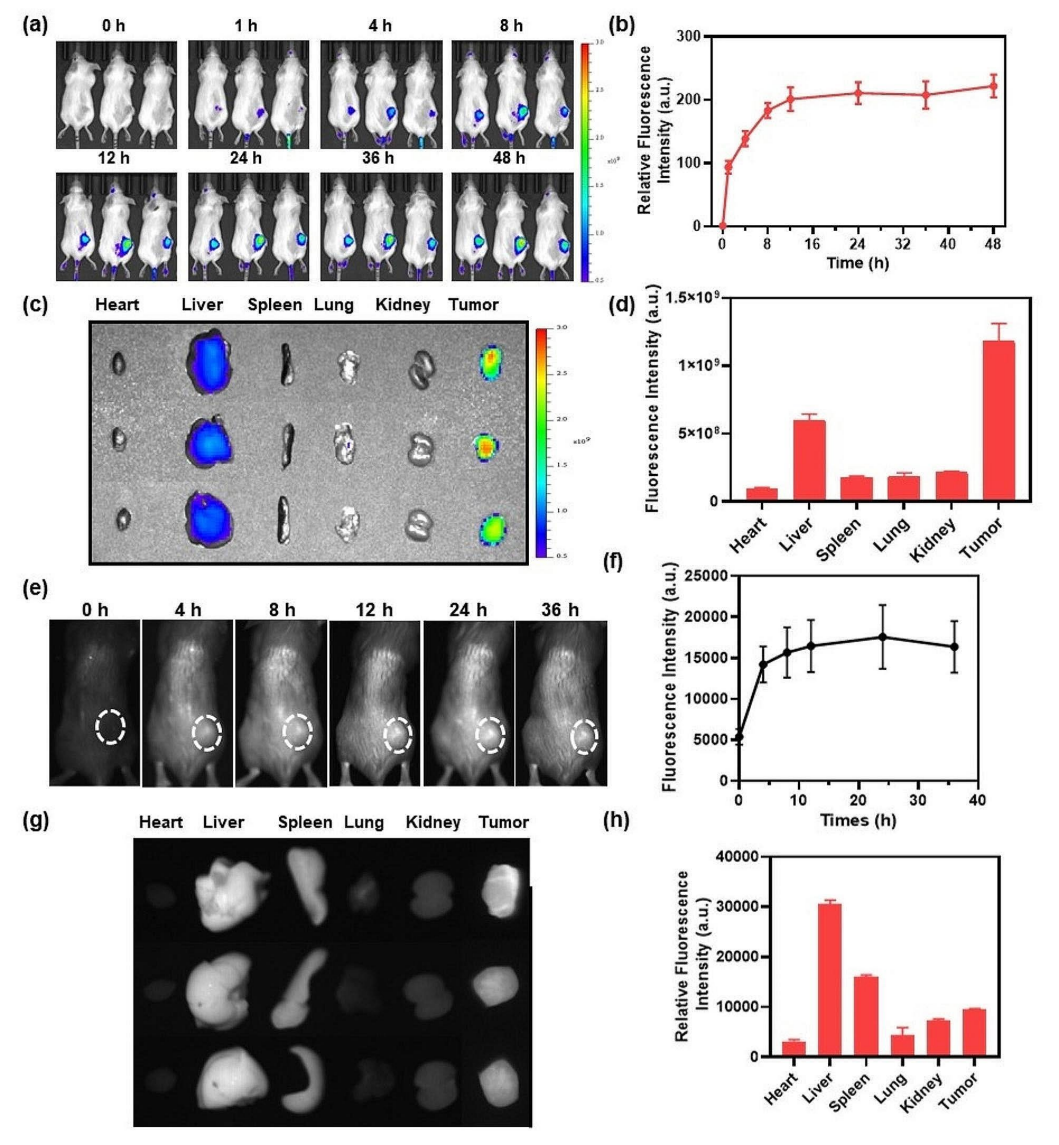
In vivo biodistribution analysis and NIR-II FLI of CR NPs. (**a**) Imaging and (**b**) quantification analysis of Colon26 tumor-bearing mice with CR/DIR NPs in different time points (*n* = 3). (**c** and **d**) Ex vivo biodistribution and quantification of the dissected main organs and tumors. (**e**) NIR-II fluorescence images and (**f**) the corresponding quantitative analysis of CR NPs in 4T1 tumor-bearing mice at different times. 4T1 tumor-bearing mice were intravenously injected with 100 µg of CR NPs (1.0 mg mL^− 1^) per mouse. Then, the real-time imaging was recorded and monitored at designated time intervals (0, 4, 8, 12, 24 and 36 h) post-injection (*n* = 3). NIR-II FLI conditions: 808 nm, 1000 nm filter, 250 ms. White dashed circles indicate tumor regions. (**g**) Ex vivo NIR-II FLI and (**h**) the corresponding quantification of major organs after 36 h administration of CR NPs

Considering the excellent in vitro properties of the CR NPs, we then studied the imaging properties of the NPs in vivo. DIR-loaded NPs (CR/DIR NPs) were constructed to monitor the in vivo biodistribution and accumulation of CR NPs using a Colon26 tumor-bearing mouse model. CR/DIR NPs were prepared using the same method as that used for the preparation of the CR NPs. When the tumor volume reached an average size of ≈ 100 mm^3^, CR/DIR NPs (50 µg ml^− 1^, 100 µL, based on DIR) were injected intravenously into the mice. The biodistribution was examined using IVIS at different time points after injection. As the whole-body images in Fig. 4a and b shown, fluorescent signals were mainly localized on tumor area and gradually enhanced over time, reaching a signal peak at 12 h after administration of CR/DIR NPs, which indicates excellent tumor accumulation of the NPs. Notably, the fluorescent signals of tumors treated with CR/DIR NPs remained clear and strong even after 48 h, which provided a long time window for PTT application. Furthermore, to evaluate the NPs distribution, ex vivo imaging of isolated main organs and tumors was conducted 48 h after the injection. As shown in Fig. 4c and d, significant fluorescence signals were still visible in the tumor and liver tissues 48 h after the injection of CR/DIR NPs, suggesting a strong passive tumor-targeting ability of CR/DIR NPs and liver clearance.

Encouraged by these results, we then evaluated the in vivo performance of CR NPs in NIR-II FLI using a 4T1 tumor-bearing BALB/c mouse model. First, the mice were intravenously administered with CR NPs (100 µL, 1 mg mL^− 1^), and the NIR-II fluorescence images were subsequently recorded at different times. In the Fig. 4e and f, the tumor profile was easily distinguishable, indicating that CR NPs could effectively accumulate at the tumor site. The NIR-II fluorescence intensities of the CR NPs at tumor regions progressively intensified within 12 h and reached the plateau at 24 h after the injection, which was in accordance with the results of NIR-I FLI (Fig. 4a and b). Therefore, the optimal time point for PTT is 12 h after the injection. Furthermore, the fluorescent signals were maintained in the tumor region for over 36 h, indicating a long therapeutic window for laser irradiation. Additionally, NIR-II ex vivo imaging of dissected tumors and vital organs was conducted 36 h after injection to further evaluate the biodistribution of the CR NPs. As shown in Fig. 4g and h, the CR NPs were mainly retained in the liver, spleen, and tumor, suggesting good tumor-targeting ability and possible metabolic organs. These imaging results demonstrate that CR NPs possess favorable tumor-targeting ability, which is conducive to tumor treatment.

Notably, these promising data demonstrate the potential for the application of CR NPs in NIR-II imaging-guided surgery. NIR-II FLI has emerged as a promising strategy for precise image-guided tumor surgery due to its deep tissue penetration and high spatial resolution. The reduced background signals, minimal tissue auto-fluorescence and low scattering in the NIR-II window enable clearer and more precise imaging for tumor margins, which is crucial for complete tumor resection while minimizing damage to surrounding healthy tissues. Several studies have shown the benefits of NIR-II FLI-guided cancer surgery, including localizing cancers, evaluating surgical margins, guiding cytoreductive surgery (CRS), tracing lymph nodes (LNs) and lymphatic vessels, and mapping specific anatomical structures. These studies have demonstrated the significant prospects of NIR FLI guided surgery in improving surgical outcomes [52]. Furthermore, the prolonged retention of CR NPs in tumor tissues provides a substantial therapeutic window, allowing surgeons sufficient time to perform image-guided surgery. Overall, CR NPs hold significant potential to improve the outcomes of surgical interventions through superb tumor targeting and imaging capabilities.

### CR NPs-mediated in vivo photothermal therapy

**Fig. 5:**
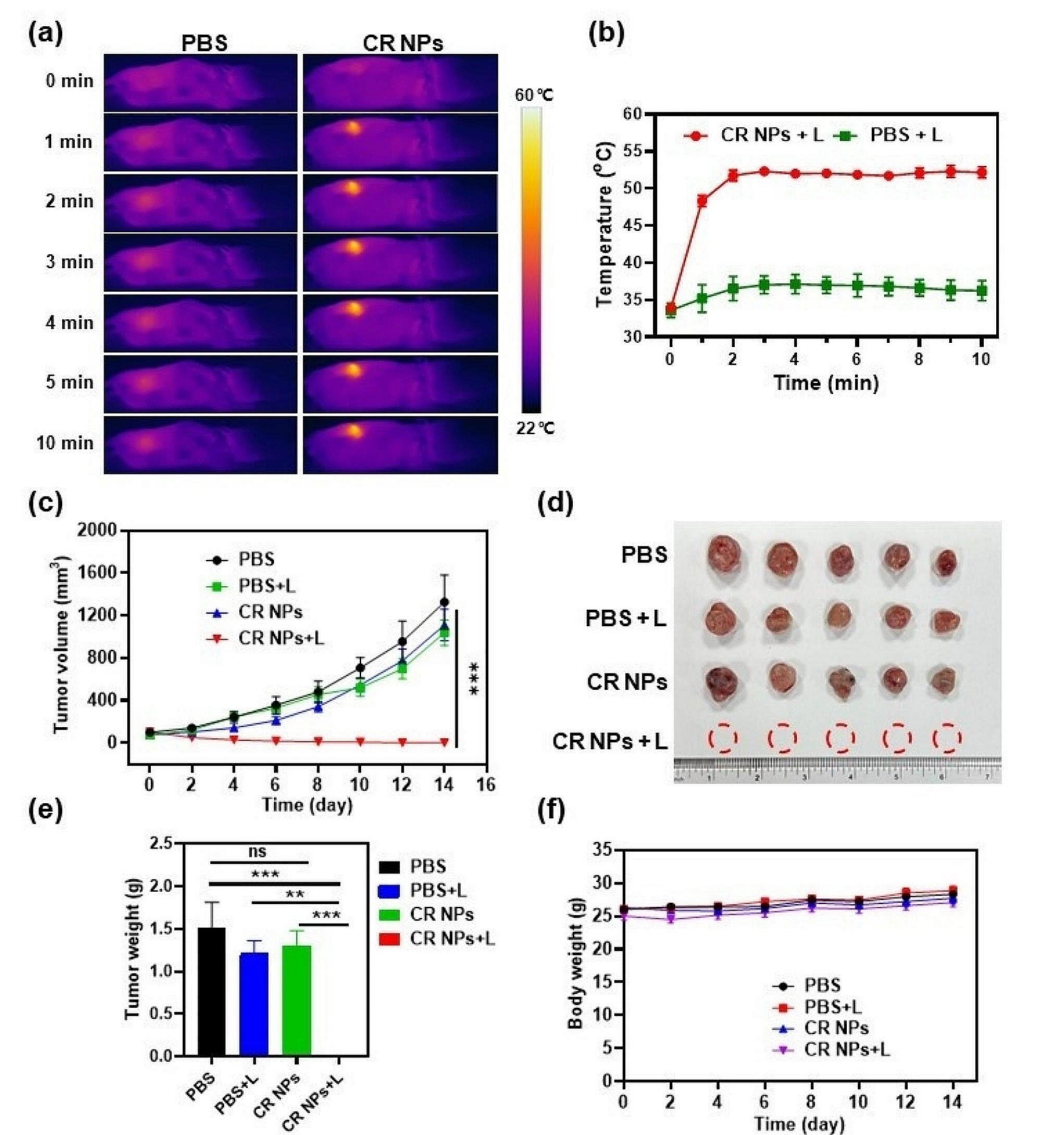
In vivo CR NPs-mediated photothermal therapy. (**a**) Infrared thermal images and (**b**) corresponding temperature profiles at the tumor sites of Colon26 tumor-bearing mice treated with PBS or CR NPs under irradiation (808 nm, 1.0 W cm^− 2^) at different time points (*n* = 5). (**c**) Tumor growth profiles of four groups during the treatment course. Statistical significance was calculated via two-way ANOVA. (**d-e**) The tumor photographs (**d**) and (**e**) weights of dissected tumors of mice in each group on the 14th day. Statistical significance was calculated via one-way ANOVA. (**f**) Body weight of mice among the treatment groups. CR NPs injection and laser irradiation were given only once

Based on the efficient in vitro PTT effect and excellent tumor accumulation of CR NPs, we then studied the PTT activity of the CR NPs in the Colon26 tumor model. When tumor size reached 100 mm^3^, the mice were randomly divided into 4 groups (5 mice per group): (1) PBS, (2) PBS + laser, (3) CR NPs, and (4) CR NPs + laser. First, the mice were intravenously injected with PBS and CR NPs; the tumors of mice in groups 2 and 4 were then continuously irradiated for 10 min 12 h post-injection according to the in vivo imaging data. The real-time photothermal images and temperatures of the mice in the laser-treated groups were recorded with an IR thermal camera to confirm the photothermal effects of the CR NPs. Figure 5a and b illustrated that the tumor region’s temperature in the CR NPs + laser treatment group rapidly increased to 52 °C within 2 min of irradiation and retained this temperature during the rest period of irradiation. The control group (PBS + laser) showed a little increase in temperature (ΔT ≈ 4 °C) under the same treatment conditions, demonstrating that the photothermal effect induced by the laser alone was negligible and did not have a PTT effect in vivo.

To further evaluate the in vivo antitumor effect of CR NPs under laser irradiation, the tumor sizes and body weights were monitored every other day after therapy. Two days after PTT, black scars were observed in the tumor area from the CR NPs + laser group, whereas insignificant changes were found in the mice from the PBS + laser group. In Fig. 5c and Figure S7, the tumors of groups (1), (2) and (3) grew continuously with similar high growth rates, and the average tumor volume increased to approximately 1500 mm^3^ within 14 days. This suggests that CR NPs or laser irradiation alone have no therapeutic effect. Notably, the NPs + laser treatment resulted in complete tumor eradication. These results indicate a significant in vivo photothermal therapeutic effect of CR NPs, which aligned with the in vitro findings. On the 14th day after treatment, the mice in all groups were sacrificed and the tumors were photographed and weighed (Fig. 5d and e). The tumor weight was in good agreement with the tendency of tumor growth profiles, further verifying the excellent in vivo photothermal ablation effect of CR NPs. The body weights of the mice were similar, with a slight increase throughout the treatment period, demonstrating satisfactory biocompatibility and limited side effects of CR NPs in vivo for PTT (Fig. 5f). Notably, a single-dose administration of CR NPs and once irradiation were conducted, which resulted in complete tumor elimination, further demonstrating the superior PTT efficacy of CR NPs for in vivo therapy.

To further evaluate the biosafety of CR NPs, histological staining of major organs and blood biochemical analysis of the treated mice were conducted at the end of treatment. As shown in Figure S8, the histological analysis of the major organs of all mice showed no significant tissue damage or inflammation after treatment, further confirming the excellent in vivo biosafety of the CR NPs. The physiological safety of the CR NPs was estimated using routine blood and biochemistry analysis (Figure S9). Various blood parameters showed no significant differences among the four groups after treatment, indicating that the CR NPs did not show systemic toxicity or harmful impacts on livers and kidneys of the mice. Taken together, CR NPs are a promising candidate for precise NIR-II FLI-guided PTT in vivo, with excellent theranostic capability and negligible side effects.

### Antitumor immune memory assessment

**Fig. 6:**
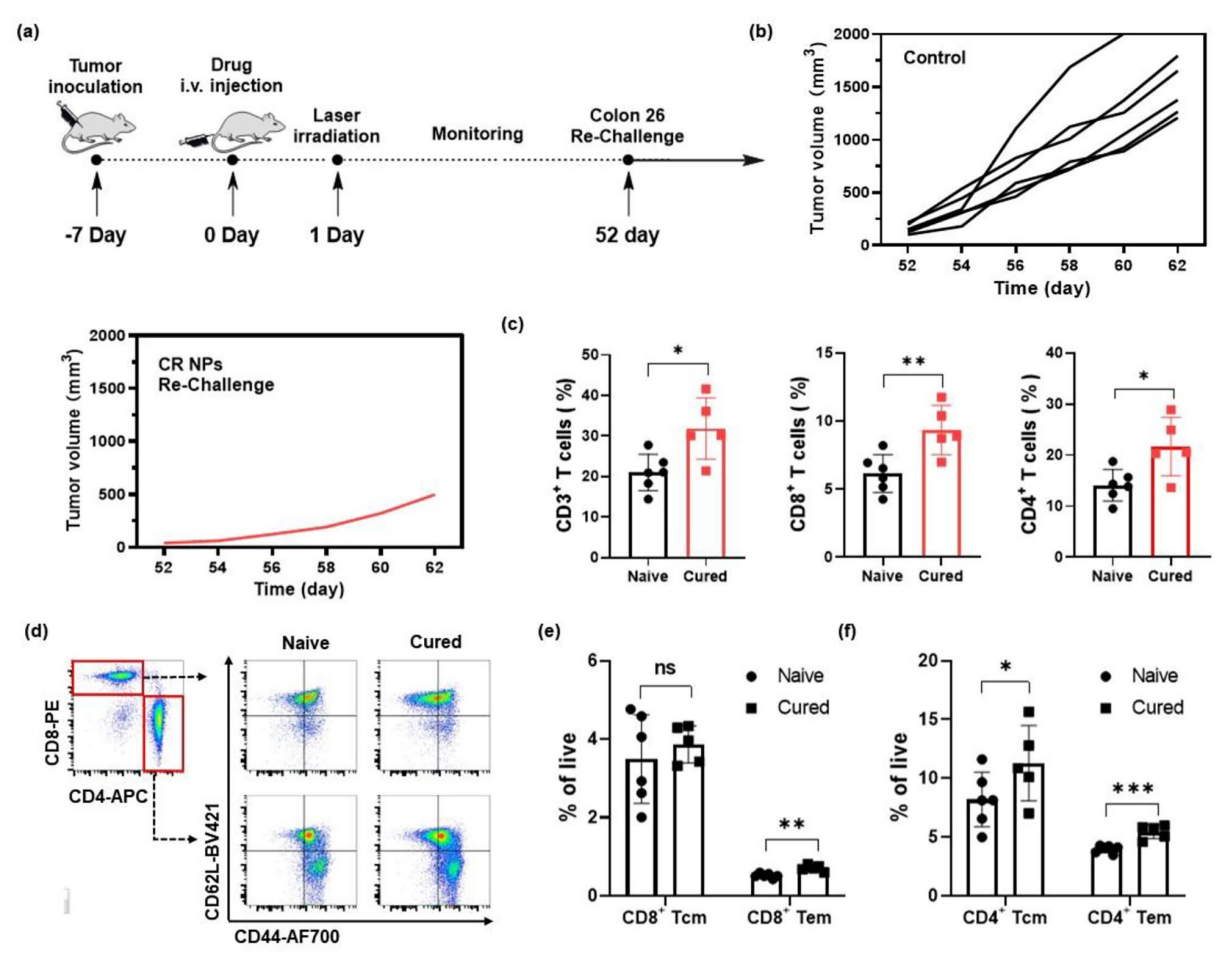
Antitumor immune memory assessment. (**a**) Mice cured by CR NPs + laser treatment were re-challenged by Colon26 cells (2 × 10^6^ cells) on day 52 after the initial treatment. (**b**) Tumor volume of each re-challenged mouse. (**c**) Quantification of CD3^+^, CD3^+^CD8^+^, CD3^+^CD4^+^ T lymphocytes in the spleen. (**d-f**) Flow cytometric (FCM) analysis and quantitative data of central memory T (Tcm, CD62L^+^CD44^+^) cells and effector memory T (Tem, CD62L^−^CD44^+^) cells subset from CD8^+^ and CD4^+^ T cells in the spleen. Data are shown as the mean ± SD, *n* = 5–6. Statistical significance was calculated via a two-tailed Student’s t-test

Given the complete tumor eradication in the Colon26 tumor model after treatment with CR NPs + laser irradiation, we wondered whether antitumor memory could be induced by CR NPs. After treatment, tumor-free mice in another batch of mice were re-challenged with 2.0 × 10^6^ Colon26 tumor cells on day 52 after the initial treatment (Fig. 6a). Age-matched mice injected with Colon26 cells (2 × 10^6^) cells were used as controls. The volumes of the tumors were measured every alternate day. As a result, the 4/5 of mice failed to form tumors in the re-challenged mice receiving the treatment of CR NPs + laser irradiation, whereas all six mice in the control group developed rapidly growing tumors (Fig. 6b), indicating that antitumor immune memory is developed after exposure to CR NPs. To elucidate the fundamental mechanism, flow cytometry was used to quantify the memory-associated T cells in the spleen. The results of Fig. 6c and Figure S10 indicated that CD3^+^, CD3^+^CD8^+^, and CD3^+^CD4^+^ T lymphocytes in the spleens of rechallenge-resistant mice were notably increased when compared with the control group. The flow cytometric analysis further revealed that effector memory T cells (Tem, CD62L^+^CD44^−^) were noticeably increased both in CD4^+^ and CD8^+^ T cells, and central memory T cells (Tcm, CD62L^+^CD44^+^) in CD4^+^ T cells were also significantly elevated (Fig. 6d–f). These results clearly indicate that the CR NPs + laser treatment can effectively inhibit tumor growth and promote the formation of long-term antitumor memory, thereby potentially preventing tumor metastasis.

### Anti-primary and metastatic tumor efficacy of CR NPs in 4T1 breast cancer model

**Fig. 7:**
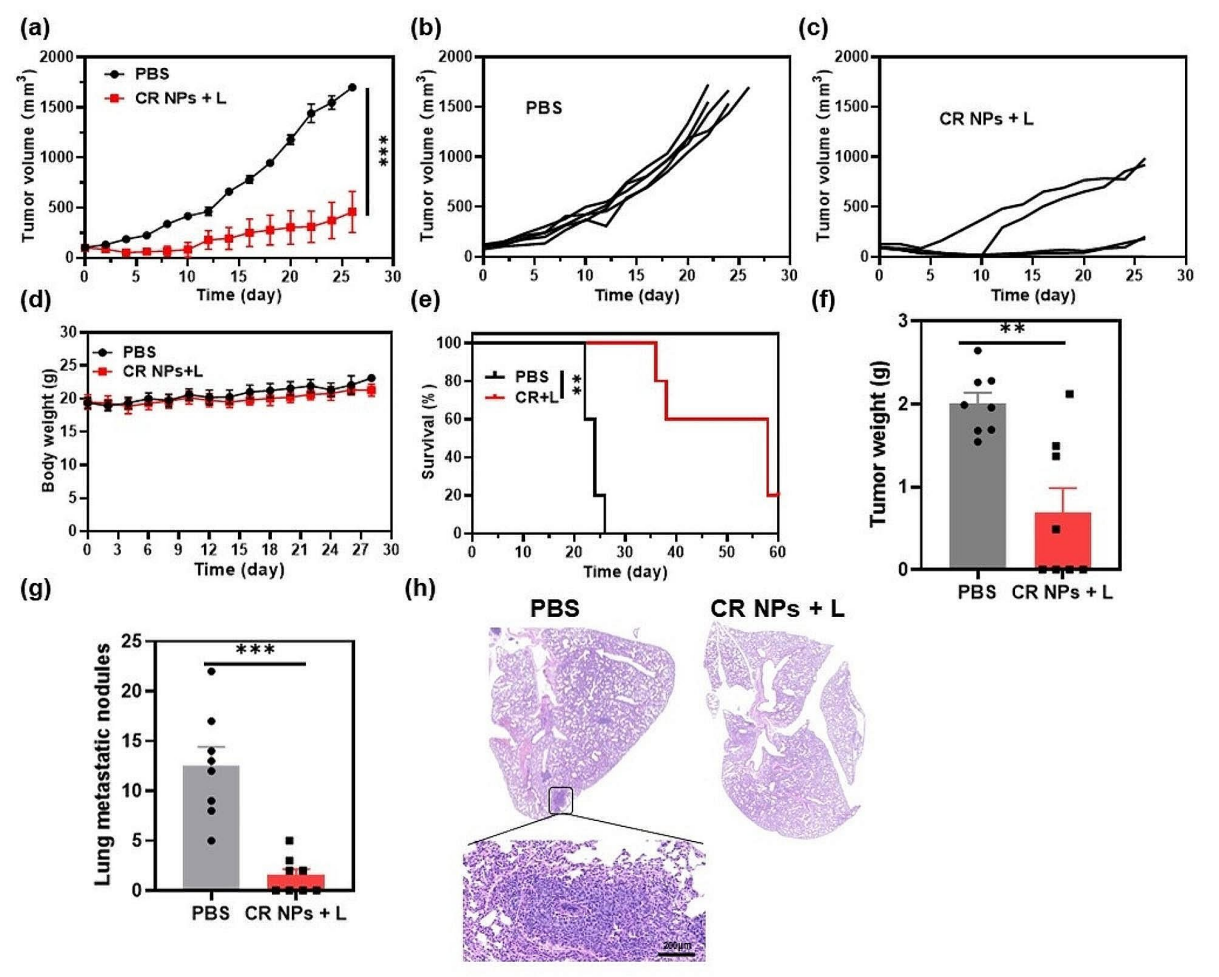
Anti-primary and metastatic tumor efficacy on a 4T1 breast cancer model by using CR NPs. (**a**) Tumor volume growth curves from the two treated groups (*n* = 5). Statistical significance was calculated via two-way ANOVA. Corresponding tumor growth curves in (**b**) PBS and (**c**) CR NPs + laser groups. (**d**) Body weights of the treated mice. (**e**) Survival curves (*n* = 5). Statistical significance was calculated via Log-rank (Mantel-Cox) test. (**f**) Tumor weights and (**g**) the number of metastatic nodules on lung surface at the endpoint. Statistical significance was calculated via unpaired Student’s-test. (**h**) Typical hematoxylin and eosin (H&E) staining of lungs from the two groups

Given the induction of long-term antitumor immune memory in Colon26 tumor-bearing mice, we further investigated whether CR NPs + laser irradiation could exert simultaneously anti-primary and anti-metastatic tumor effects using an orthotopic 4T1 murine mammary carcinoma model which could induce lung metastasis during primary tumor development. We first assessed the anti-primary tumor performance in this refractory tumor model. When the tumor volumes reached 100 mm^3^, the mice were treated with PBS or CR NPs + laser irradiation 12 h after injection. The tumor volumes and body weights were recorded every other day during the treatment period. The results showed that CR NPs + laser irradiation effectively inhibited orthotopic 4T1 tumor growth, although no tumor eradication was observed owing to its resistance to therapy (Fig. 7a–c), demonstrating the wide applicability of CR NPs-mediated PTT in different tumor types. Similarly, CR + laser treatment did not result in any significant alterations in animal body weight compared to PBS treatment (Fig. 7d). The survival time of the mice in the CR NPs + laser treatment group was also greatly prolonged (Fig. 7e), whereas tumors in mice treated with PBS alone grew rapidly, with an average lifespan of only 20–26 days. These results suggest that CR NPs + laser irradiation is highly efficient and safe for the in vivo photothermal ablation of highly metastatic tumors.

As cancer metastasis is the main cause of tumor-associated death, we subsequently investigated whether CR NPs + laser irradiation could reduce lung metastasis of 4T1 tumors after treatment in another batch of tumor-bearing mice. Tumor growth of mice in the CRs + laser group was greatly inhibited, which was consistent with the results of the first batch of mice (Figure S11). The tumors and lungs of the mice were collected on day 30 after treatment to evaluate the antimetastatic effects. As displayed in Fig. 7f and g, the primary tumor weight was significantly lower in CR NPs + laser-treated mice than in control mice, and the number of pulmonary metastatic tumor nodules on the lung surface was also remarkably reduced in treated mice when compared with the control group. The lungs were sectioned for histological evaluation, and almost no metastatic lesions were observed in the CR NPs + laser-treated group (Fig. 7h), whereas significant metastatic foci were discovered in the control group’s lungs, indicating that CR NPs-based PTT effectively inhibits the lung metastasis of tumor cells. Therefore, the CR NPs-based PTT can induce a potent systemic immune response and prevent tumor metastasis.

## Conclusions

In short, we reported an NIR-II emissive nanoparticle (CR NPs) from PEGylated pure organic small molecules (CR-TPE-T) and demonstrated NIR-II FLI-guided PTT for cancers. The synthesized CR NPs displayed prominent photothermal conversion capability, satisfactory photothermal stability, high biocompatibility, and efficient passive tumor targeting and accumulation. In vitro experiments indicated that CR NPs had negligible dark toxicity and highly efficient photocytotoxicity toward tumor cells, making them promising for PTT application. The NIR-II FLI of CR NPs enabled real-time monitoring of the tumor location and growth with high accuracy, and confirmed the ideal PTT window after the injection. Additionally, CR NPs achieved excellent in vivo PTT efficacy and achieved complete tumor ablation in the Colon26 mouse model, and induced long-term immune memory, which was triggered by a single dose of NIR irradiation. Furthermore, antitumor assessment in refractory 4T1 bearing-tumor mice revealed that CR NPs efficiently inhibited tumor growth, improved the survival of mice, and induced efficient systemic immune responses, which further reduced lung metastasis. Systemic administration of CR NPs did not show signs of toxicity or side effects, thus providing important therapeutic benefits. To our knowledge, this work is the first report of NIR-II-emissive SOPTAs based on croconaine dye, therefore provides a significant advancement in NIR-II nanomedicine. Overall, this study demonstrates a superior photothermal agent for NIR-II imaging-guided PTT with impressive tumor fluorescence imaging capability, efficient therapeutic functions, and noticeable biosafety. We believe that this study will open up new avenues for the construction of NIR-II-emitting SOPTAs for cancer phototheranostics, which is crucial for improving the clinical application of PTT.

### Electronic supplementary material

Below is the link to the electronic supplementary material.

Supplementary Material 1

## Data Availability

No datasets were generated or analysed during the current study.
