# Supplementary material for: Croconaine-based NIR-II fluorescence imaging-guided tumor photothermal therapy induces long-term antitumor immune memory
Source: J Nanobiotechnology. 2024 Aug 13;22:481. doi: 10.1186/s12951-024-02695-y (PMC11321165; doi:10.1186/s12951-024-02695-y)
Supplement: Supplementary file 1 — Supplementary Material 1 [file 12951_2024_2695_MOESM1_ESM.docx]

**Croconaine-Based NIR-II Fluorescence Imaging-Guided Tumor Photothermal Therapy Induces Long-Term Antitumor Immune Memory**

Yafang Dong^1, 2, #^, Huifang Wang^1, #^, Youbin Ding^3^, Xiaodong Zhang^3^, Yucheng Zou^2^, Zhijie Li^1, *^, Shan-Chao Zhao^2, 4, 5, *^, Jigang Wang^1, 2, 6, 7, 8, 9, *^

^1^Department of Urology, Shenzhen People’s Hospital, The First Affiliated Hospital, Southern University of Science and Technology, Shenzhen 518020, Guangdong, PR China

^2^Department of Urology, the Third Affiliated Hospital of Southern Medical University, Guangzhou, 510500, Guangdong, P. R. China.

^3^Department of Medical Imaging, The Third Affiliated Hospital of Southern Medical University, Guangzhou, 510630, Guangdong, P. R. China.

^4^Department of Urology, the Fifth Affiliated Hospital, Southern Medical University, Guangzhou, 510500, Guangdong, P. R. China.

^5^Department of Urology, Nanfang Hospital, Southern Medical University, Guangzhou, 510515, Guangdong, P. R. China.

^6^Department of Traditional Chinese Medicine, School of Pharmaceutical Sciences, Southern Medical University, Guangzhou, Guangdong 510515, PR China.

^7^State Key Laboratory for Quality Ensurance and Sustainable Use of Dao-di Herbs, Artemisinin Research Center, and Institute of Chinese Materia Medica, China Academy of Chinese Medical Sciences, Beijing 100700, P. R. China.

^8^State Key Laboratory of Antiviral Drugs, School of Pharmacy, Henan University, Kaifeng 475004, China.

^9^Department of Oncology, the Affiliated Hospital of Southwest Medical University, Luzhou, Sichuan, P. R. China.

^#^These authors equally contribute to this work and share the first authorship.

*Corresponding authors:

jgwang@icmm.ac.cn (Jigang Wang);

li.zhijie@szhospital.com (Zhijie Li);

lulululu@smu.edu.cn (Shanchao Zhao)

**General information**

Croconic acid, n-butanol, toluene, and DMSO were commercially available and used as received without further purification unless otherwise specified. N,N-bis(4-(1,2,2-triphenylvinyl)phenyl)thiophen-2-amine (**S1**) and **CR-TPE-T** were synthesized according to the reported protocol [1]. Colon 26 and 4T1 cells were obtained from Cell Bank of the Chinese Academy of Sciences (Shanghai, China), Roswell Park Memorial Institute (RPMI) 1640 medium, fetal bovine serum (FBS), Exosome-depleted Fetal Bovine Serum, penicillinstreptomycin (Pen-Strep), and Lipofectamine™ 3000 Transfection Reagent were obtained from Thermo Fisher Scientific (Waltham, MA, USA). Cell Counting Kit-8 (CCK-8) was purchased from Dojindo Laboratories (Kumamoto, Japan). Annexin V-FITC/PI apoptosis kit was obtained from MultiSciences (Hangzhou, China). Apoptosis detection assay was analyzed using a flow cytometer (CytoFLEX; Beckman Coulter, Inc.) and the data were analyzed using FlowJo software (FlowJo, LLC). Anti-CALR (Abcam, ab92516) and anti-HMGB1 (Abcam, ab18256), anti-mouse CD16/CD32 antibody (BioXcell, BE0307) CD45-BV570 (Biolegend, 103136), CD3-FITC (BD Biosciences, 553062), CD11b-APC-Cy7 (Biolegend, 101226), CD4-APC (Biolegend, 100412), CD8a-PE (Biolegend, 100708), CD62L-BV421 (Biolegend, 104435), and CD44-AF700 (Biolegend, 103026). Confocal laser scanning microscopy (CLSM) characterization was carried out on a confocal laser scanning microscope (TCS SP8, Leica, Germany). Paraformaldehyde (PFA) (4%) was purchased from Biyuntian Company (Shanghai, China). Cell membrane NIR fluorescent probe DIR was purchased from Xi’an Ruixi Biological TechnologyCo., Ltd. Slide-A-Lyzer Mini Dialysis devices were supplied from Repligen Corporation (Waltham, MA, USA). Utra-100K was purchased from Millipore (Billerica, MA, USA). All reactions were carried out under dry nitrogen by using Schlenk techniques. Reaction progress was monitored by thin-layer chromatography (TLC) on silica plates (250 µmol/L thickness, bought from Qingdao Haiyang Chemical Co.) and spots were visualized by UV254 and 365 fluorescent indicators. Flash column chromatography was carried out using silica gel (100 mesh) bought from Aladdin.

UV-Vis-NIR absorption spectra were determined at RT (room temperature) upon 201 UV-visible spectrophotometers (Thermal Fisher Scientific, USA) with 1nm resolution, through quartz cuvettes (1cm path-length). The fluorescence spectrum was recorded on a Hitachi F-4500 fluorimeter equipped with a Xenon lamp excitation lamp (λ_ex_ = 808 nm, Spectrofluorometer FS5, Edinburgh).

**Synthesis of CR-TPE-T** [1]

**3,5-Bis(5-(bis(tetraphenylethylene)amino)thiophen-2-yl)cyclopentane-1,2,4-trione (CR-TPE-T)**. **S1** (0.76 g, 1.0 mmol, 2.0 eq), croconic acid (0.071 g, 0.5 mmol, 1.0 eq) were added into a 50 mL two-necked round-bottom flask with magnetic stirrer and a reflux condenser. The flask was degassed and flushed with nitrogen three times. Sequentially, 20 mL mixture of toluene/1-butanol (v/v, 1/1) was injected into the flask, and the reaction mixture was refluxed at 130 ^o^ C for 24 h under nitrogen atmosphere. Afterwards, the solvent was evaporated, and the crude product was purified by silica-gel column chromatography by using DCM/methanol (200:1 to 100:1) as eluent to give CR-TPE-T (0.163 g) in 20% yield.

**Computational details**

The theoretical calculations were performed via the Gaussian 16 suite of programs [1]. The structure of the studied molecule was fully optimized at the B3LYP-D3BJ/6-31G* level of theory. The vibrational frequencies of the optimized structures were carried out at the same level. The structures were characterized as a local energy minimum on the potential energy surface by verifying that all the vibrational frequencies were real. The molecular orbital levels of studied compounds were investigated via theoretical calculations, including the highest occupied molecular orbital (HOMO) and the lowest unoccupied molecular orbital (LUMO). The Visual Molecular Dynamics (VMD) program [1] was used to plot the color-filled iso-surface graphs to visualize the molecular orbitals.

**Supplementary figures**

**Scheme S1.** Synthetic scheme of **CR-TPE-T**.

**
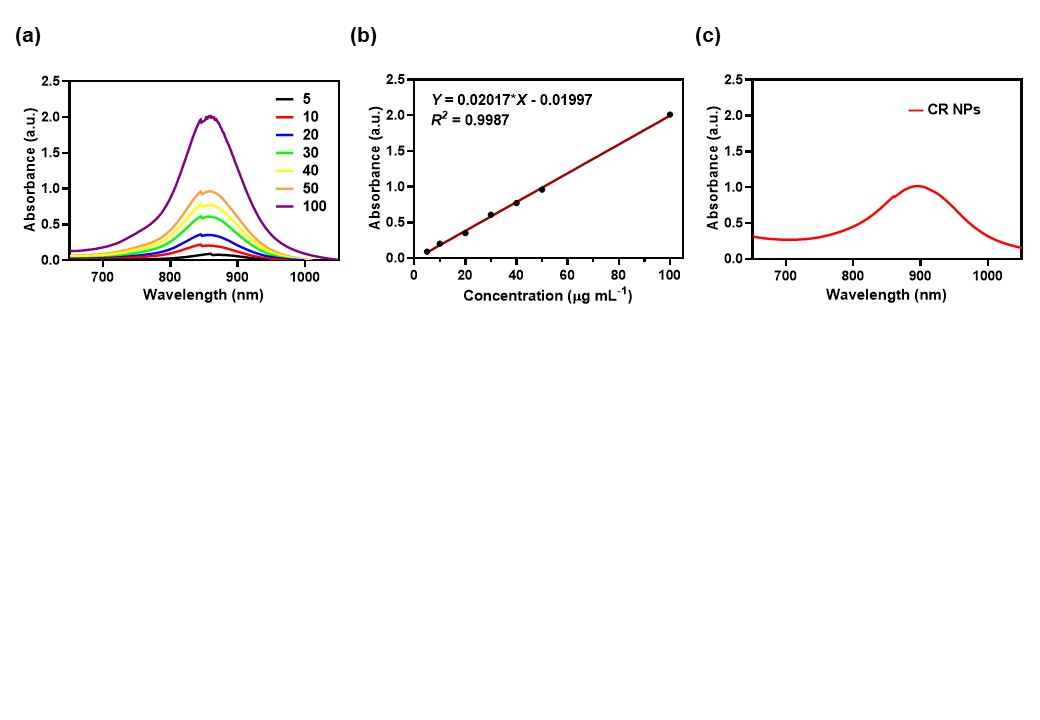
**

**Figure S1.** Loading efficiency determination of CR molecule into nanoparticles (NPs). (a) UV-Vis spectra of CR in THF versus varying concentrations (5-100 µg mL^−1^). (b) Calibration curve of absorption vs. concentration of CR in THF at 860 nm. (c) Absorption spectrum of the above prepared CR NPs dissolved in THF (diluted 5 times).

**
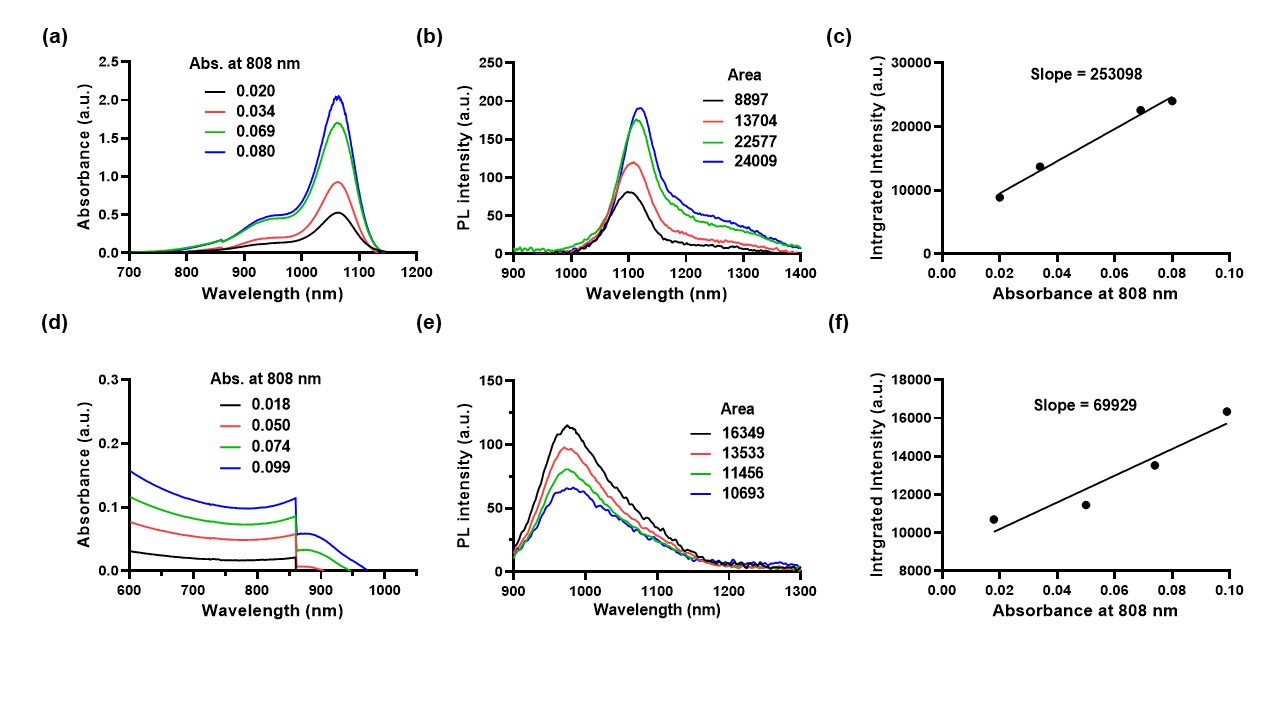
Figure S2.** Determination of fluorescence quantum yield of CR NPs. (a) UV-Vis-NIR spectra of IR-1061 in DCM at different concentrations. (b) NIR emission spectra of IR-1061 in DCM at different concentrations under 808 nm excitation. The area under curve (AUC) in the emission spectra for each solution was calculated and listed on the right of the graph. (c) For all IR-1061 DCM solutions, their absorbance values were plotted versus AUC, and fitted into a linear function. (d) UV-Vis-NIR absorption spectra of CR NPs in water at different concentrations. (e) NIR emission spectra of CR NPs in water at different concentrations under 808 nm excitation. AUC in the emission spectra for each solution was calculated and listed on the right of the graph. (f) For all CR NPs solutions, their absorbance values were then plotted versus AUC, and fitted into a linear function.


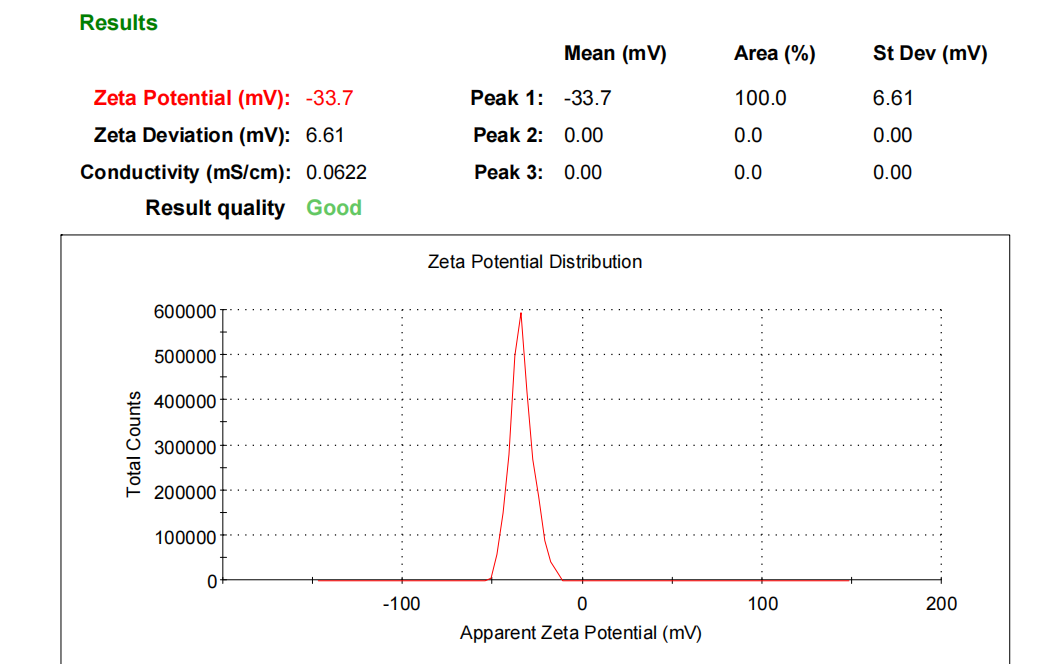


**Figure S3.** Zeta potential of the CR NPs in deionized water.

**
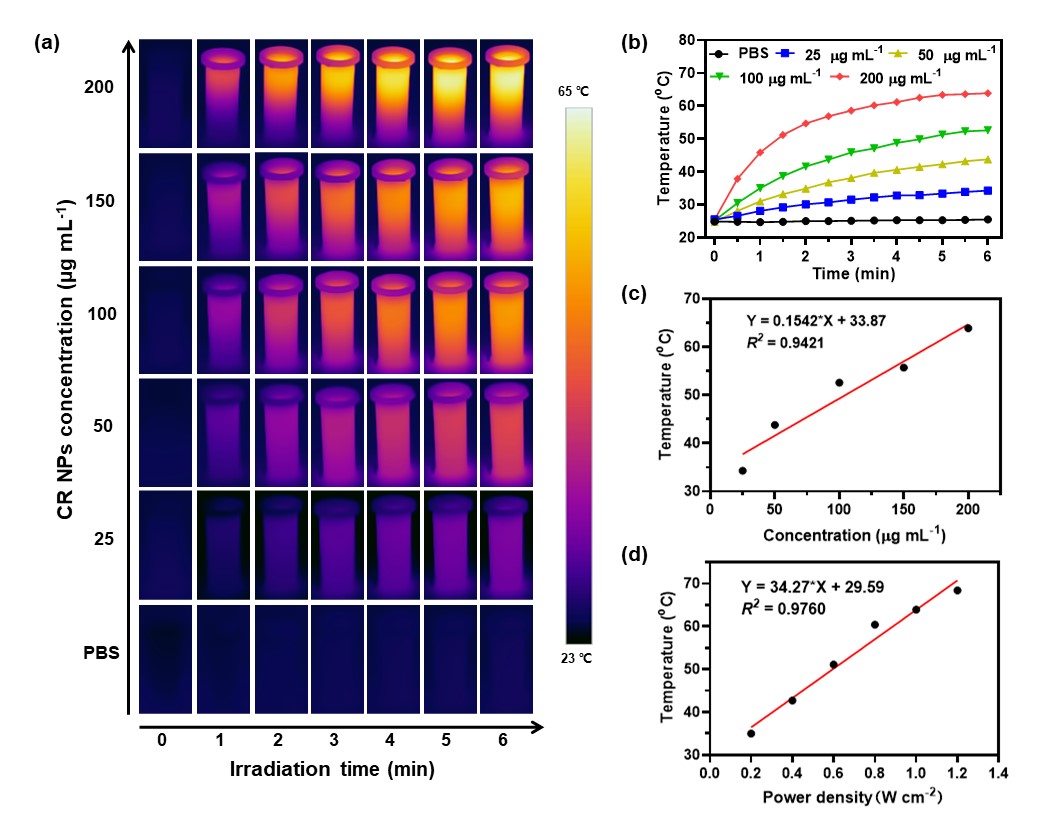
Figure S4.** (a) Real-time thermal imaging and (b) photothermal conversion behavior of CR NPs under 808 nm laser illumination with different concentration (0–200 µg mL^−1^) for 6 min. Photothermal behavior shows a proportional relationship with (c) the nanoparticle concentration and (d) laser power density.

**
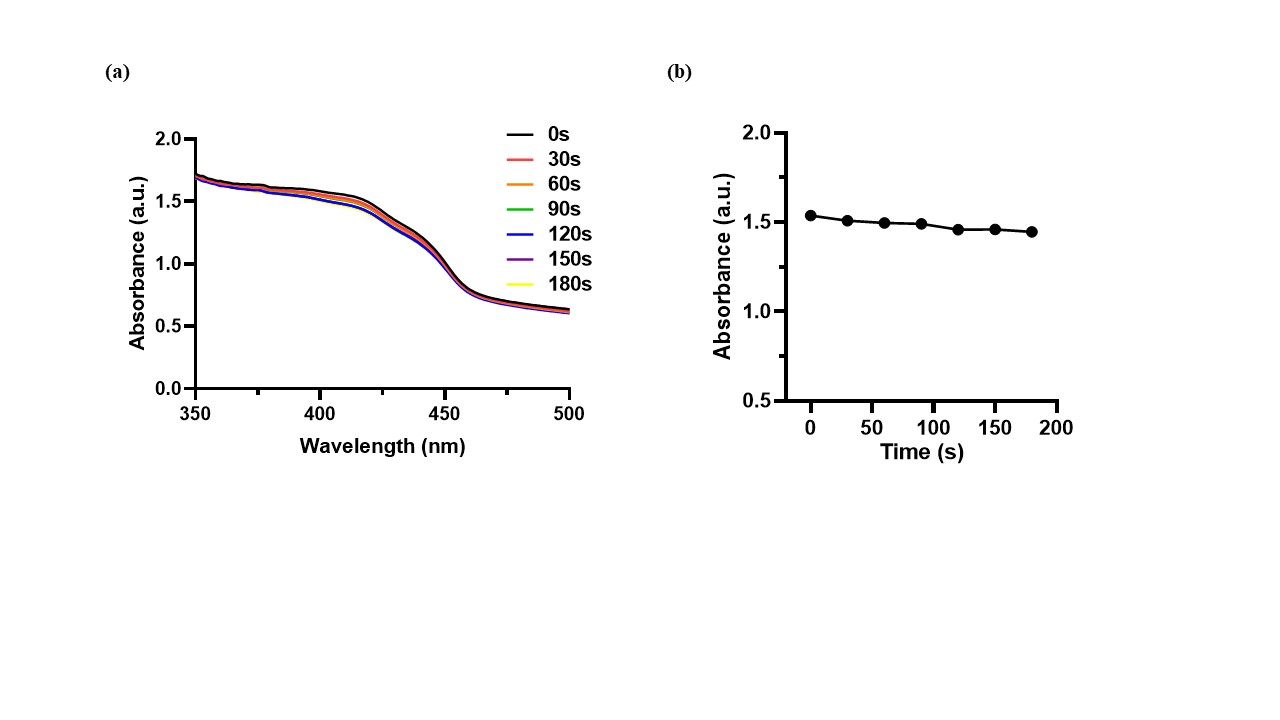
Figure S5.** Detection of singlet oxygen production by CR NPs with 808 nm laser irradiation. (a) Absorption spectra of the mixed aqueous solution of CR NPs and DPBF under 808 nm laser irradiation (1 W cm^−2^) over time. (b) Absorbance at 414 nm of DPBF solution in the presence of CR NPs in the indicated period.

**
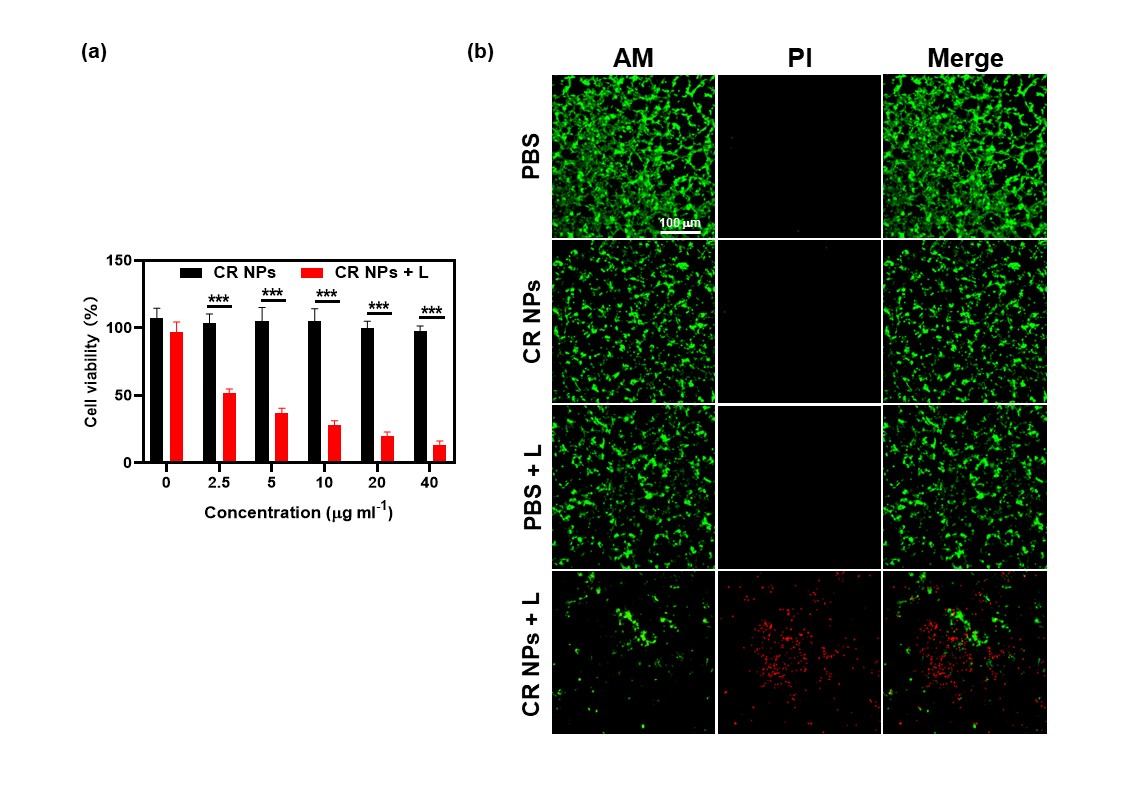
Figure S6.** (a) Cytotoxicity of 4T1 cells treated with various concentrations of CR NPs, with or without laser irradiation (808 nm, 1.0 W cm^−2^, 5 min), as determined using a CCK-8 assay. n = 6. (b) Live/dead cell analysis after different treatments with or without NIR irradiation in Colon 26 cells through Calcein-AM (green) and PI (red) staining recorded by confocal fluorescence microscopy imaging. (Scare bars, 100 µm).


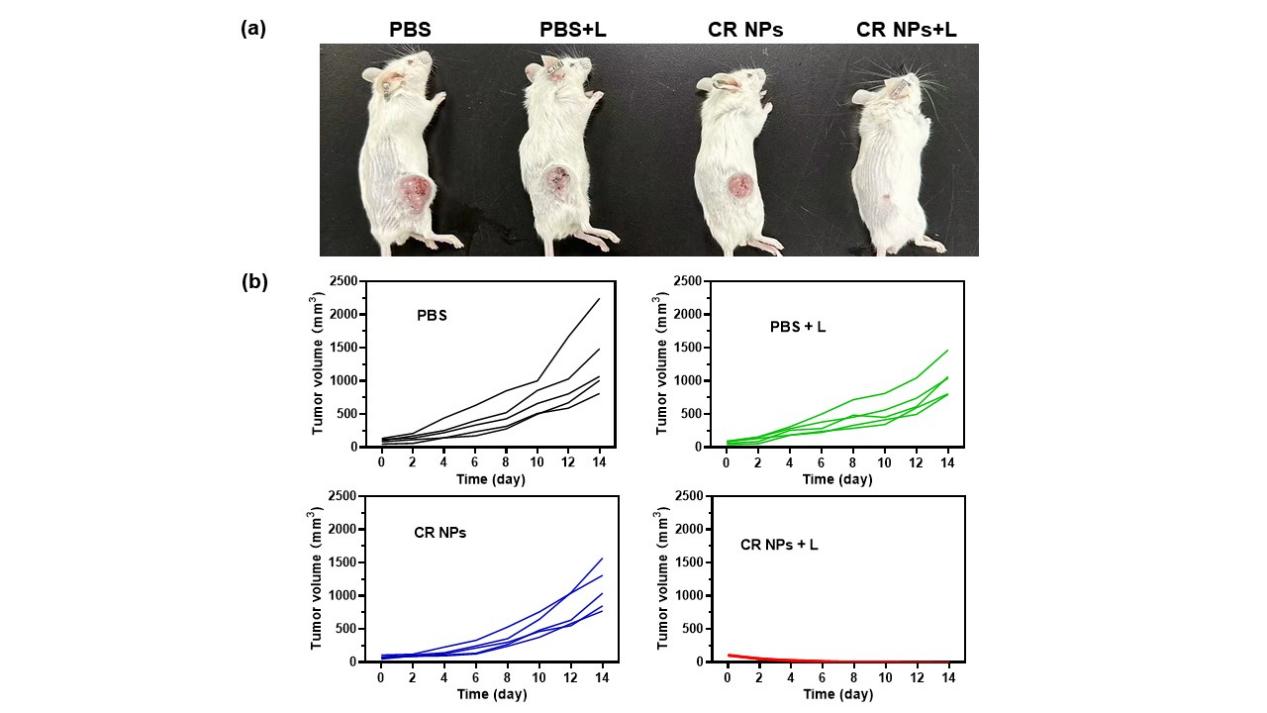
**Figure S7.** (a) Representative images of Colon 26 tumor-bearing mice taken on day 14 post-treatment and (b) the corresponding individual tumor growth curve.


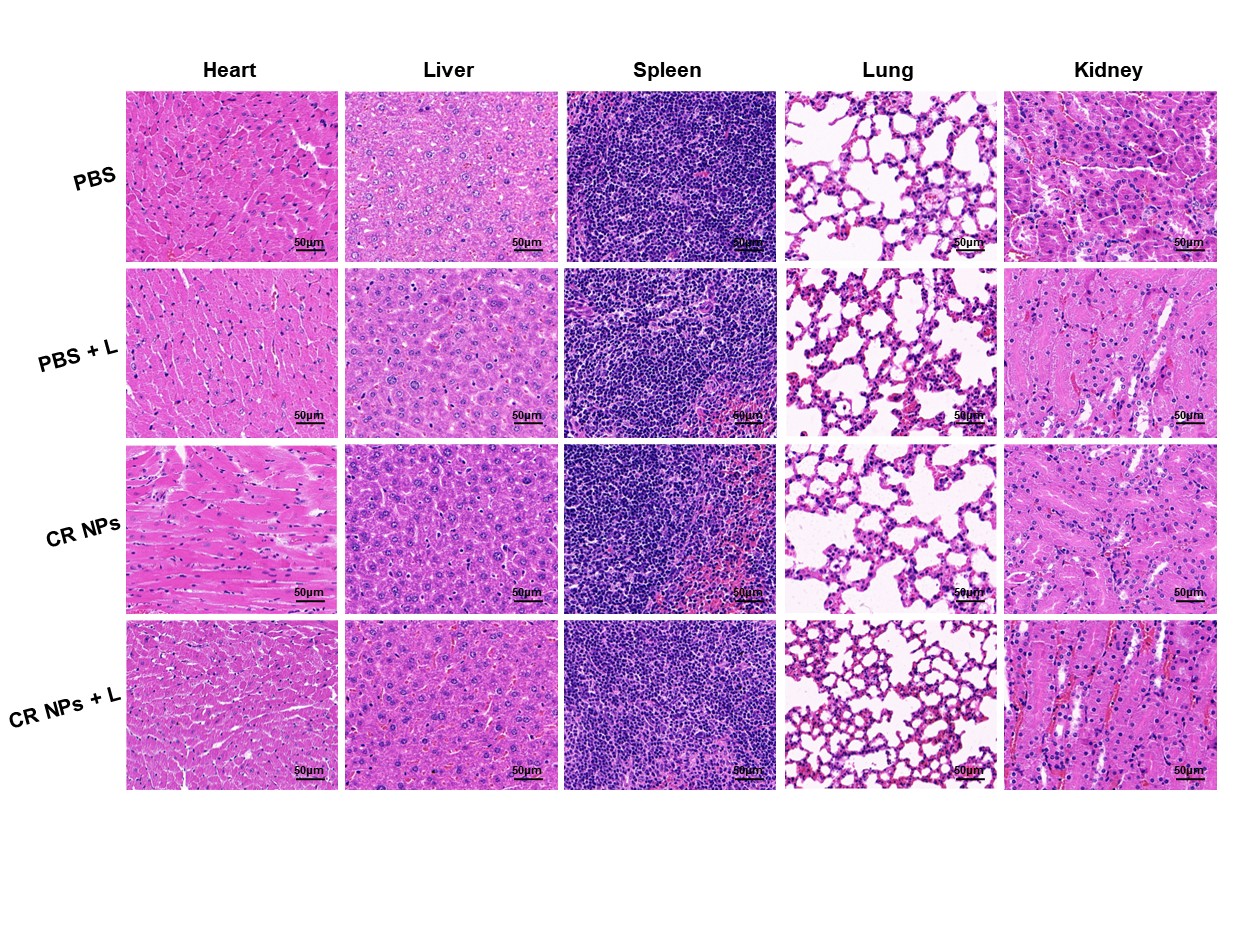
**Figure S8.** H&E staining of major organs (heart, liver, spleen, lung, kidney) of mice in each group. Scale bars, 50 μm.


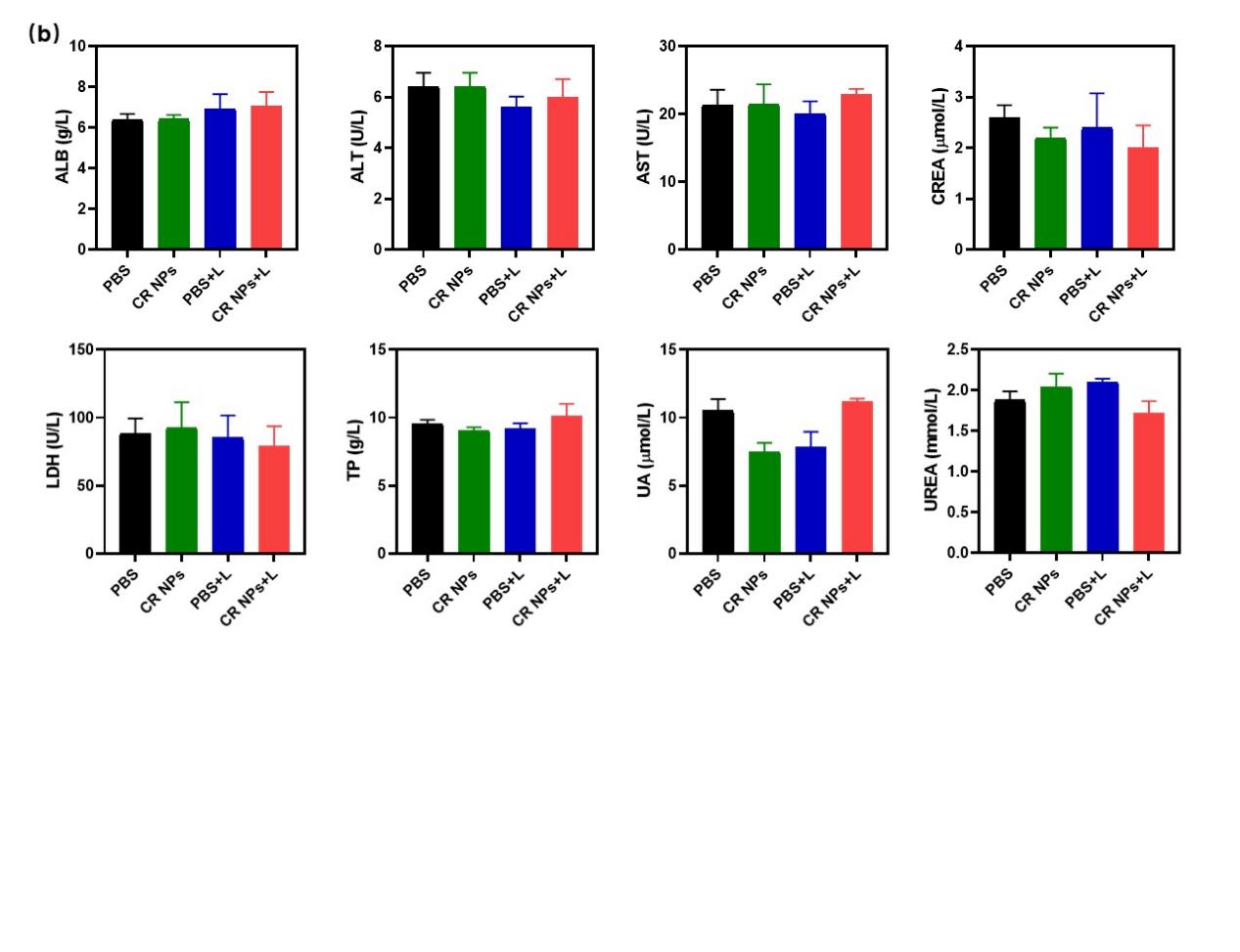

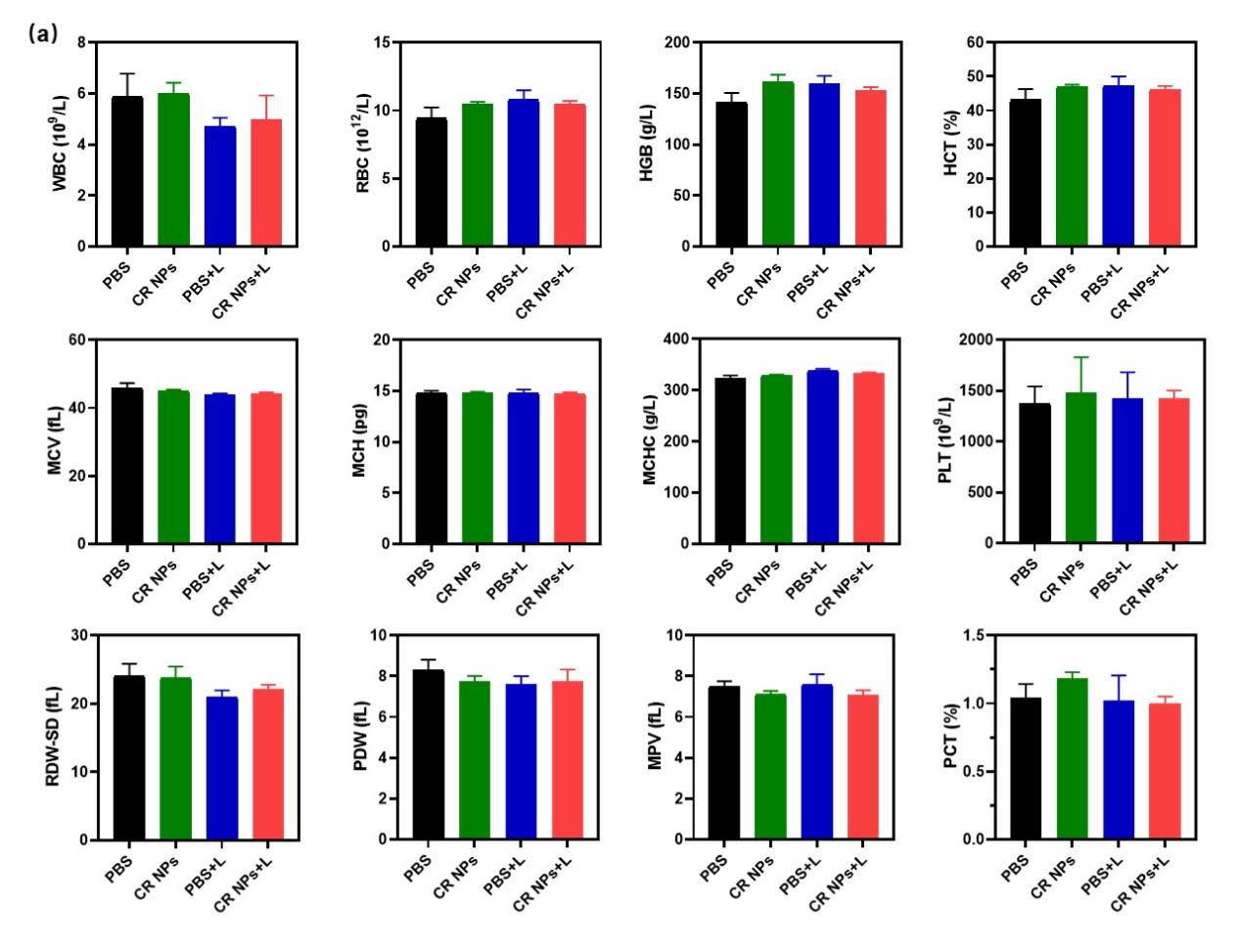
**Figure S9. (a)** The complete blood analysis and **(b)** Blood biochemistry analysis of different groups 14 days post-injection (n = 5).

**Figure S10.** Flow cytometric (FCM) analysis of CD3^+^, CD3^+^CD8^+^, CD3^+^CD4^+^ T lymphocytes in the spleen for memory T cell assessment**
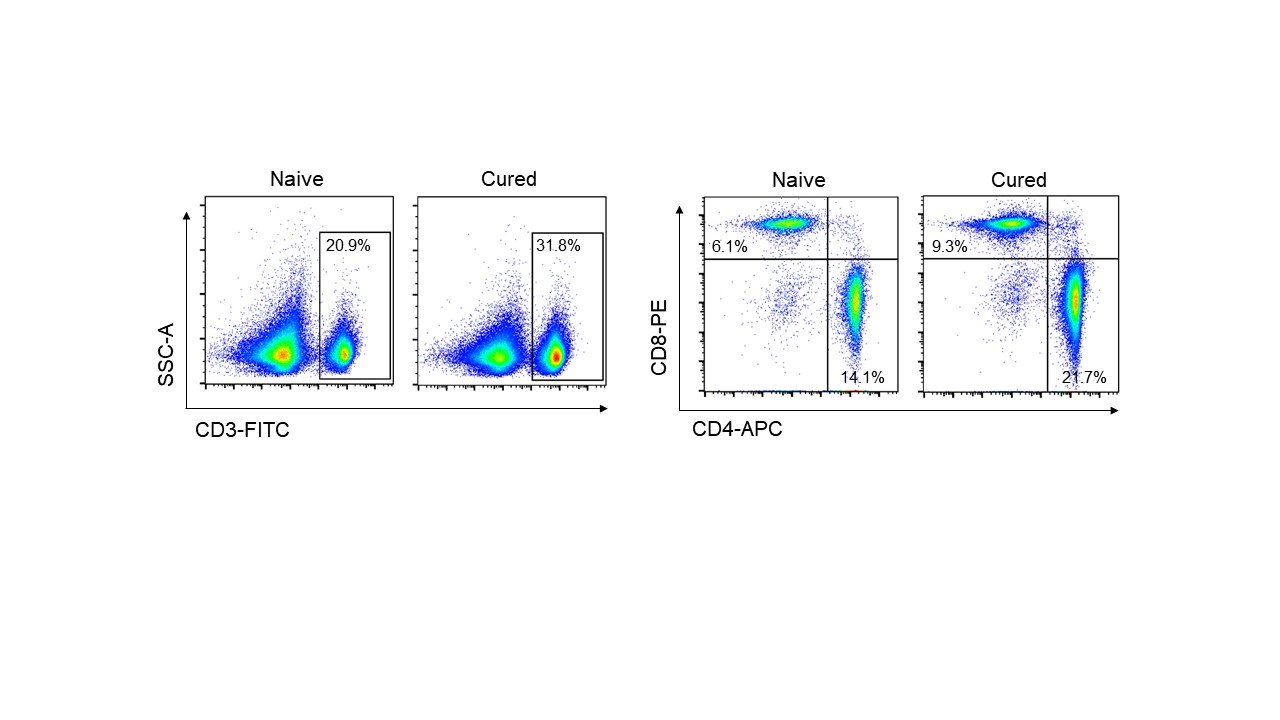
.**


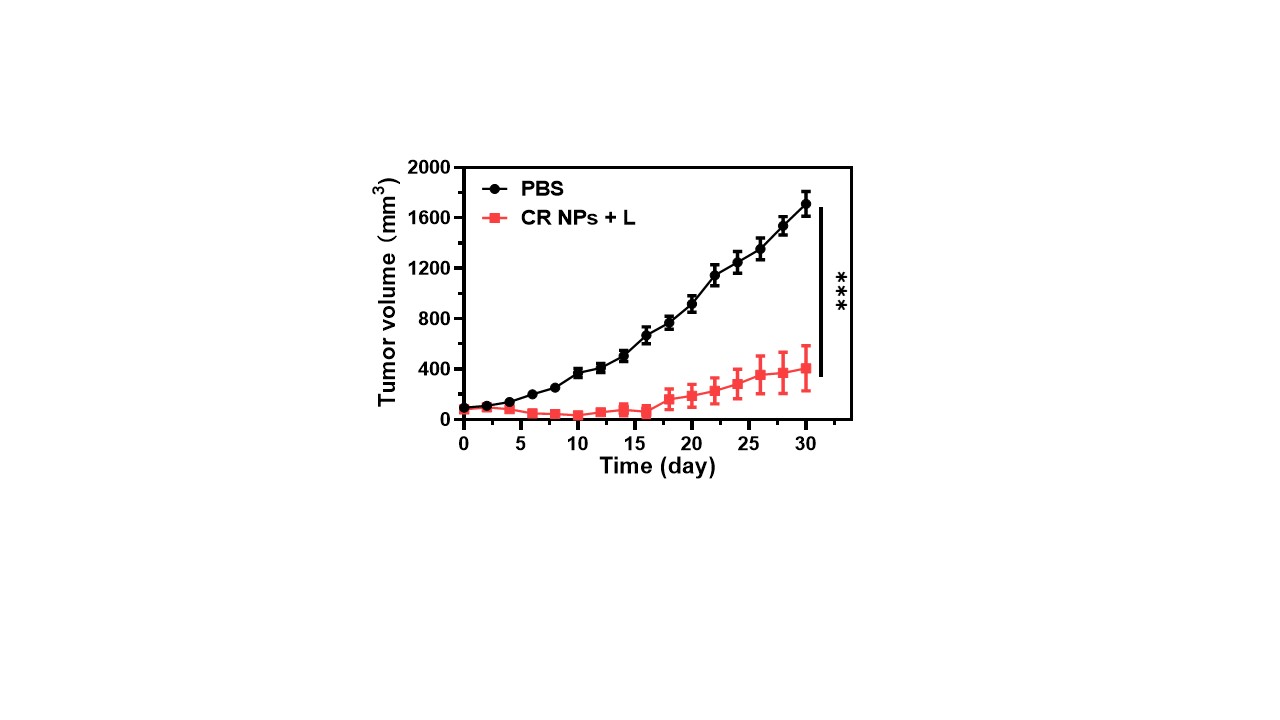


**Figure S11.** Tumor growth curves from the orthotopic breast tumor-bearing mice in 2nd batch of mice (n = 8 mice per group).

**References**

[1] Chen G, Sun J, Peng Q, Sun Q, Wang G, Cai Y, Gu X, Shuai Z, Tang BZ. Biradical-featured stable organic-small-molecule photothermal materials for highly efficient solar-driven water evaporation. Adv Mater. 2020;32:e1908537.

[2] Frisch MJ, Trucks GW, Schlegel HB, Scuseria GE, Robb MA, Cheeseman JR, Scalmani G, Barone V, Petersson GA, Nakatsuji H, et al. Gaussian 16 Revision. A 03, Gaussian Inc. Wallingford, CT, 2016.

[3] Humphrey W, Dalke A, Schulten K. VMD: Visual molecular dynamics. J Mol Graph Model 1996;14:33–8.
